# Spatial localization of sound elicits early responses from occipital visual cortex in humans

**DOI:** 10.1038/s41598-017-09142-z

**Published:** 2017-09-05

**Authors:** Claudio Campus, Giulio Sandini, Maria Concetta Morrone, Monica Gori

**Affiliations:** 1 U-VIP Unit for Visually Impaired People, Fondazione Istituto Italiano di Tecnologia, Via Morego, 30-16163 Genoa, Italy; 2 RBCS Robotics, Brain and Cognitive Sciences dept., Fondazione Istituto Italiano di Tecnologia, Via Morego, 30-16163 Genoa, Italy; 3 Department of Translational Research on New Technologies in Medicine and Surgery, University of Pisa, via San Zeno 31, 56123 Pisa, Italy; 4 Scientific Institute Stella Maris IRCCS, Calambrone, Pisa, Italy

## Abstract

Much evidence points to an interaction between vision and audition at early cortical sites. However, the functional role of these interactions is not yet understood. Here we show an early response of the occipital cortex to sound that it is strongly linked to the spatial localization task performed by the observer. The early occipital response to a sound, usually absent, increased by more than 10-fold when presented during a space localization task, but not during a time localization task. The response amplification was not only specific to the task, but surprisingly also to the position of the stimulus in the two hemifields. We suggest that early occipital processing of sound is linked to the construction of an audio spatial map that may utilize the visual map of the occipital cortex.

## Introduction

Space representation is one hard problem that the brain has to face, given the continuously changing flow of information collected by our mobile sensors, for review see refs 1–3. Of all the sensory systems, vision relies on the representation of space from orderly retinotopic maps. It is not clear to date whether this sophisticated retinotopic code of the early visual cortex can also be used to derive the spatial position of other sensory sources, for example for the localization of a sound. Sound localization in space is bound to visual maps in many structures of the monkey brain. The acoustic receptive fields (RFs) of the neurons of the inferior colliculus, as well as of the Superior Temporal Sulcus, are eye-centered and move with the eye^2, 4–8^, but no data indicate that this also occurs in the primary cortex; or more generally, that the occipital visual cortex’s response to sound may be important for the construction of metrics aimed at representing sounds in. A neuronal response of primary the visual cortex to sound alone has been demonstrated in mice^9^, in cats^10^, in primates^4, 11, 12^, and crucially also in humans, by fMRI^13–16^, TMS^17^ and ECoG^18^ and EEG^19^ studies. Several anatomical routes that may mediate the auditory response in the occipital cortex have been identified, like a direct thalamo-occipital pathway^20^ or a cortico-cortical pathway that feeds back the auditory cortical response to the visual cortex through the parietal cortex^12, 21^. The acoustic projections in monkeys are also heterogeneously distributed across the retinotopic representations within the primary visual cortex, with more peripheral visual field representations receiving denser projections^11, 21^.

Despite the demonstration of neuronal activity and pathways in animal models, and the clear evidence from fMRI, previous studies using scalp Event Related Potentials (ERP) in sighted humans have failed to demonstrate a reliable early response specifically involving striate areas to a unimodal auditory stimulation^22, 23^. The lack of such activity was observed both in detection^19, 24^ and in discrimination tasks^25^. Recent ERP studies have shown an auditory-evoked contralateral occipital activation (ACOP) in sighted individuals^22, 23, 26, 27^. ACOP pattern revealed that the cross-modal activations of visual cortices by sounds were critically dependent on whether the sound location was predictable or not, indicating that this particular cross-modal process is not (fully) automatic; instead, it is context-contingent^26^. However, this response was characterized by estimated sources localized to the ventrolateral extrastriate visual cortex (Brodmann’s area 19) and by a late time window, between 250 and 400 ms after the sound.

In contrast to the paucity of studies showing early visual cortical response to sound alone in normal human subjects, clear evidence demonstrates a neuronal interaction between vision and audition in early visual cortex, for example an amplification/reduction of the visual response to a congruent/incongruent audio-visual stimuli^13, 19, 24, 25, 28, 29^. All these data demonstrate that the visual cortex plays an important role in audio-visual integration^17, 30–35^. Early visual cortex can even encode the abstract information of the acoustic object shape^14^, clearly demonstrating that the visual cortex plays a role in acoustic perception. Furthermore, animal literature suggests that early visual coding may be important for solving the spatial localization of sound^4, 9–12, 16^.

It has been shown that first involvement of visual cortex in the processing of auditory stimuli occurs shortly after sensory stimulation: ECoG recordings highlighted first neural markers within 28 ms of sound offset^18^, while scalp EEG showed first ERP modulations around 50 ms^19^. Therefore, we hypothesized the existence, in this purely auditory bisection task, of an ERP component which 1) is specifically elicited by a task involving the development of a spatial metric; 2) occurs in an early time window involved in multisensory integration.

In our experiment we measured an early cortical response to sound alone in normal blindfolded subjects during a spatial localization task and compared the response to the same acoustic stimuli measured during a temporal localization task. This response occurs in an early time window (50–90 ms), which plays a key role in the earliest stages of multisensory integration. Moreover, the observed early component mimics many characteristics of the C1 ERP component normally elicited by visual stimuli and is specifically elicited during a spatial, but not during temporal bisection task, even though the two tasks presented identical stimuli.

## Results

### Sensor Level Analysis

**Figure 1:**
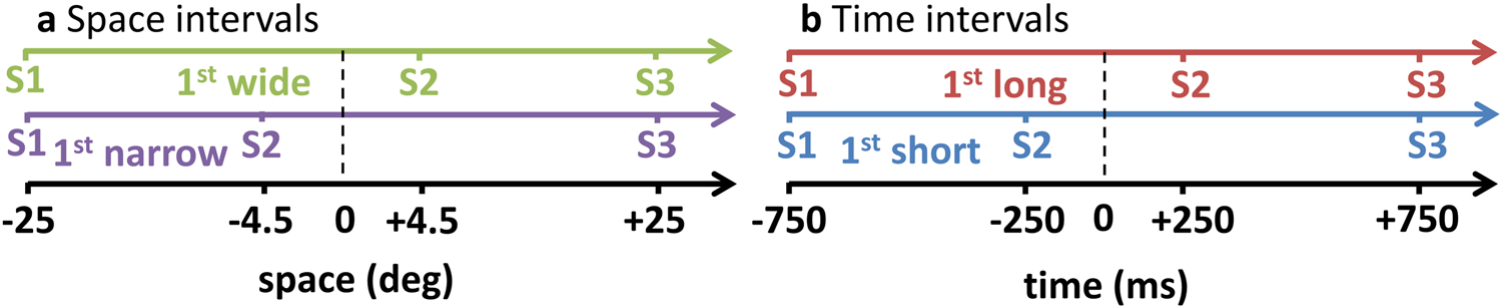
Setup for auditory spatio-temporal bisection. For each trial, subjects listened to a sequence of 3 sounds in the lower visual hemifield (S1, S2, S3) that lasted 1.5 sec with S1 and S3 at fixed position of ±25° with respect to the subject midline. S2 could occur randomly and independently at ±4.5° **(a)** and ±250 ms **(b)** from the physical spatial and temporal mid points (dashed vertical line). To avoid stereotypical subject responses, S2 was also presented at 0° and at 0 ms during catch trials. Subjects were instructed to judge whether the position of S2 in space or in time (two separate blocks) is farther from S1 or S3 (bisection task).

16 blindfolded naïve subjects performed an auditory bisection task (Fig. 1) to a sequence of 3 sounds provided in the lower visual hemifield, where the middle sound could be delivered at two different spatial positions and two different temporal lags independently. The separation of the second sound from the physical bisection position was close to threshold for the spatial and temporal tasks, assessed in a preliminary study on a separate group of subjects (mean ± SEM spatial bisection thresholds equal to 4^o^ ± 2 ^o^ and temporal bisection thresholds 250 ± 15 ms). Subjects were asked whether the first interval was temporally longer and whether it was spatially larger than the second one.

**Figure 2:**
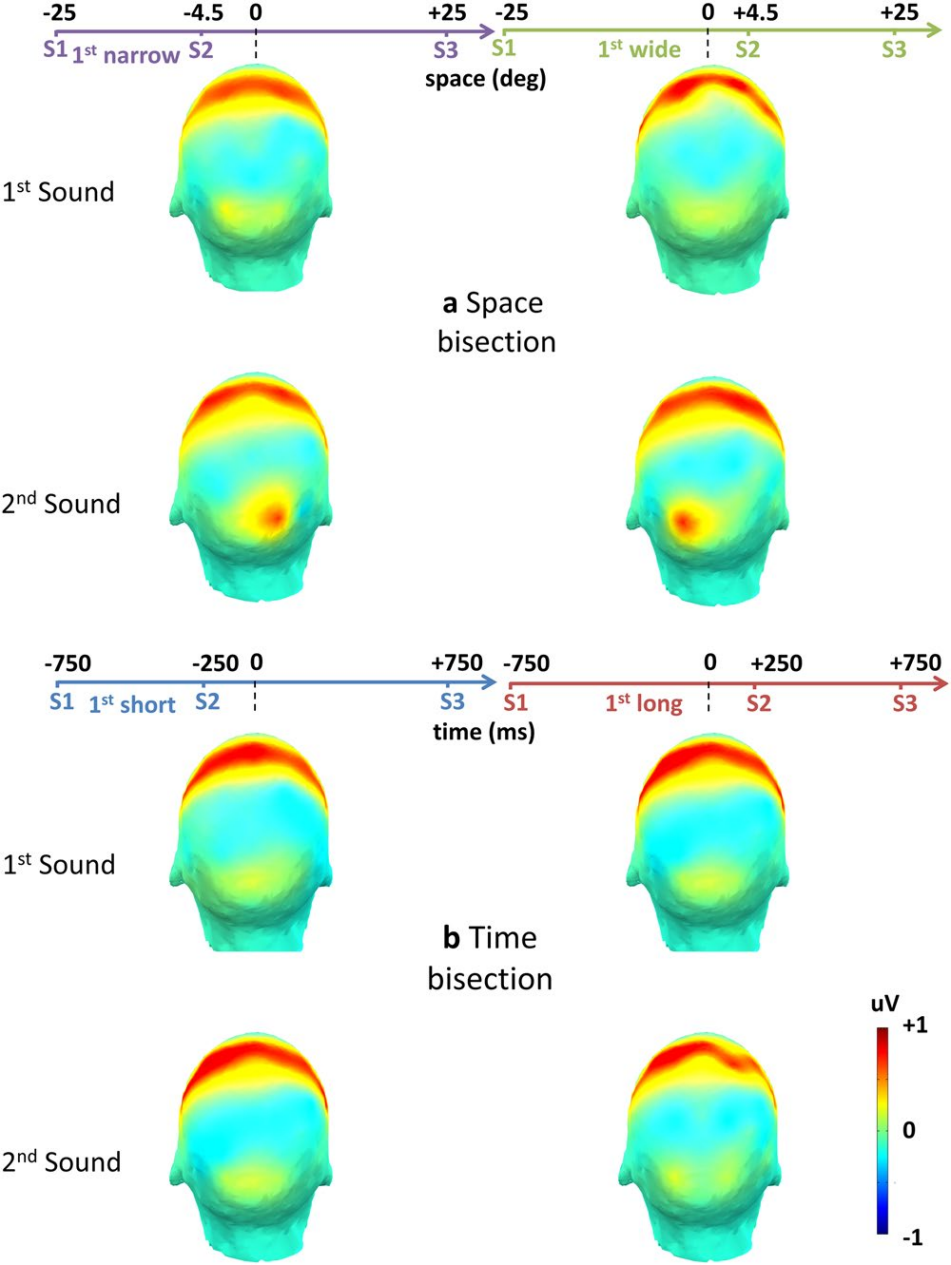
ERP scalp map in the (50–90 ms) time window averaged across subjects. Two strong positivities emerged. One, involving central areas, was not modulated by experimental manipulation. The other, involving parieto-occipital areas, showed a specific contralaterality during the space bisection task (**a**) when a narrow (Left) or a wide (Right) first interval respectively corresponded to a second sound in the left or in the right hemifield. During the time bisection task (**b**), neither a short nor a long first interval in the time domain could elicit a similar response. The observed contralateral response was specific to the second sound, while absent after the first one. Importantly, no other scalp areas were involved in early positivity.

Here we report event related potentials (ERP) in response to the first sound, considered as a control, and to the second sound, considered as the starting point for the development of a metric. Mean ERP amplitude was computed by averaging the voltage in the early occipital ERP component time windows. Evaluated time window (50–90 ms) and scalp topography (O1, O2, C1, C2 electrodes) were based on the grand average obtained by merging all conditions (see Supplementary Fig. 1 and Fig. 2 respectively).

ANOVA on mean ERP amplitude in the selected time window revealed a significant interaction between AREA (Central, Occipital), HEMISPHERE (Left, Right), FIRST INTERVAL EXTENSION (Small, Large), DOMAIN INVOLVED IN BISECTION TASK (Space, Time) and SOUND (First, Second), F(1,15) = 47.3, P = 0.000005, generalized eta squared = 0.29^36^.

**Figure 3:**
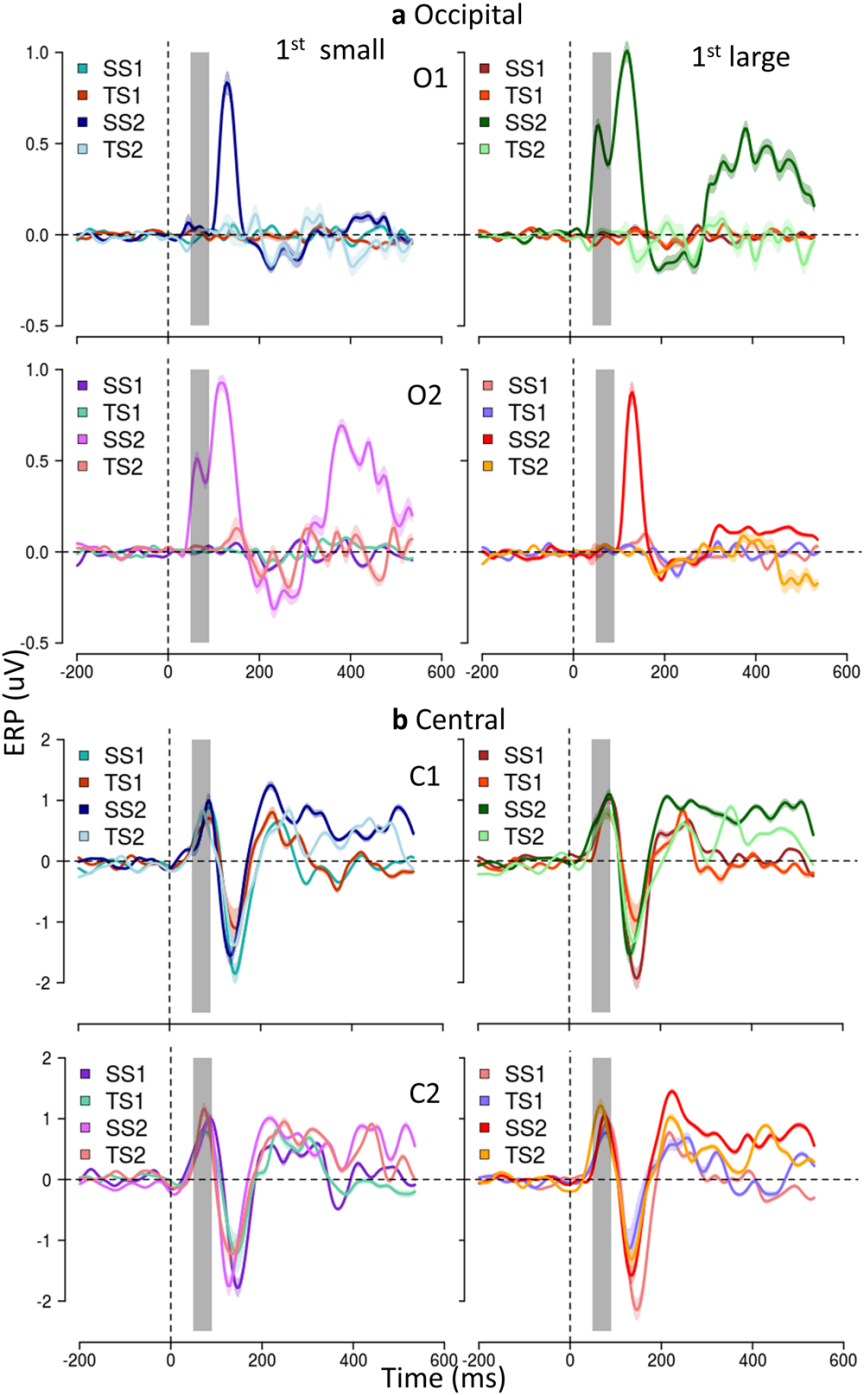
Physical position of the second sound modulates early response in Occipital but not in Central areas. (**a**) Occipital areas. ERP (mean ± SEM) in O1, first raw, and in O2, second raw, averaged across subjects. On the left, average of trials in which S2 is presented in the left hemispace (spatial bisection) or with shorter temporal separation from S1 (temporal bisection); on the right, trials in which S2 is presented in the right hemispace or with longer temporal separation from S1. Differently colored curves represent ERP responses to the first and the second sound for temporal (TS1, TS2) and spatial bisection task (SS1, SS2). t = 0 is sound onset. The shaded area delimits early ERP component time window (50–90 ms), commonly corresponding to major visual responses. (**b**) ERP in C1, first line, and in C2, second line (mean ± SEM). Only in Occipital areas both the early and a later response (starting around 350 ms after the sound) show a contralateral pattern with respect to the second sound and during the spatial bisection task.

Figure 2 reports the scalp topography of mean ERP amplitude in the (50–90 ms) time window and Fig. 3 reports the ERP response to the first and the second sounds, the second being presented at ±4.5^°^ in space independently of the presentation time (±250ms).

A prominent positivity was found in both Central and Occipital areas, but only the latter showed a specific contralateral pattern with respect to the second sound and only during spatial bisection task (compare Fig 2a and b, as well as the left and right columns of Fig. 3 in the dashed areas).

Although the temporal separation between the first two sounds is very large (0.75 ± 0.1 sec) to allow a complete decay of the ERP response, the occipital component in the 50–90 ms time window was about four times larger for the second sound than for the first (mean ± SEM amplitude for S1 = 0.06 ± 0.04µV, for S2 = 0.46 ± 0.1µV, t_15_ = 3.61, P = 0.003). However this amplification was present only during the spatial bisection task, and not during the temporal task (S1 = 0.07 ± 0.06 µV, S2 = 0.08 ± 0.07 µV, t_15_ = 0.12, P = 0.91).

Note that the stimuli in the two conditions were identical, with the same spatial and temporal separation, the only difference being the task of the subjects.

Importantly, the selected time window is the first one showing a modulation due to the task, while a much later modulation seems to occur in agreement with an auditory-evoked contralateral occipital activation (ACOP) previously observed in sighted individuals between 250 and 400 ms^22, 23, 26, 27^.

**Figure 4:**
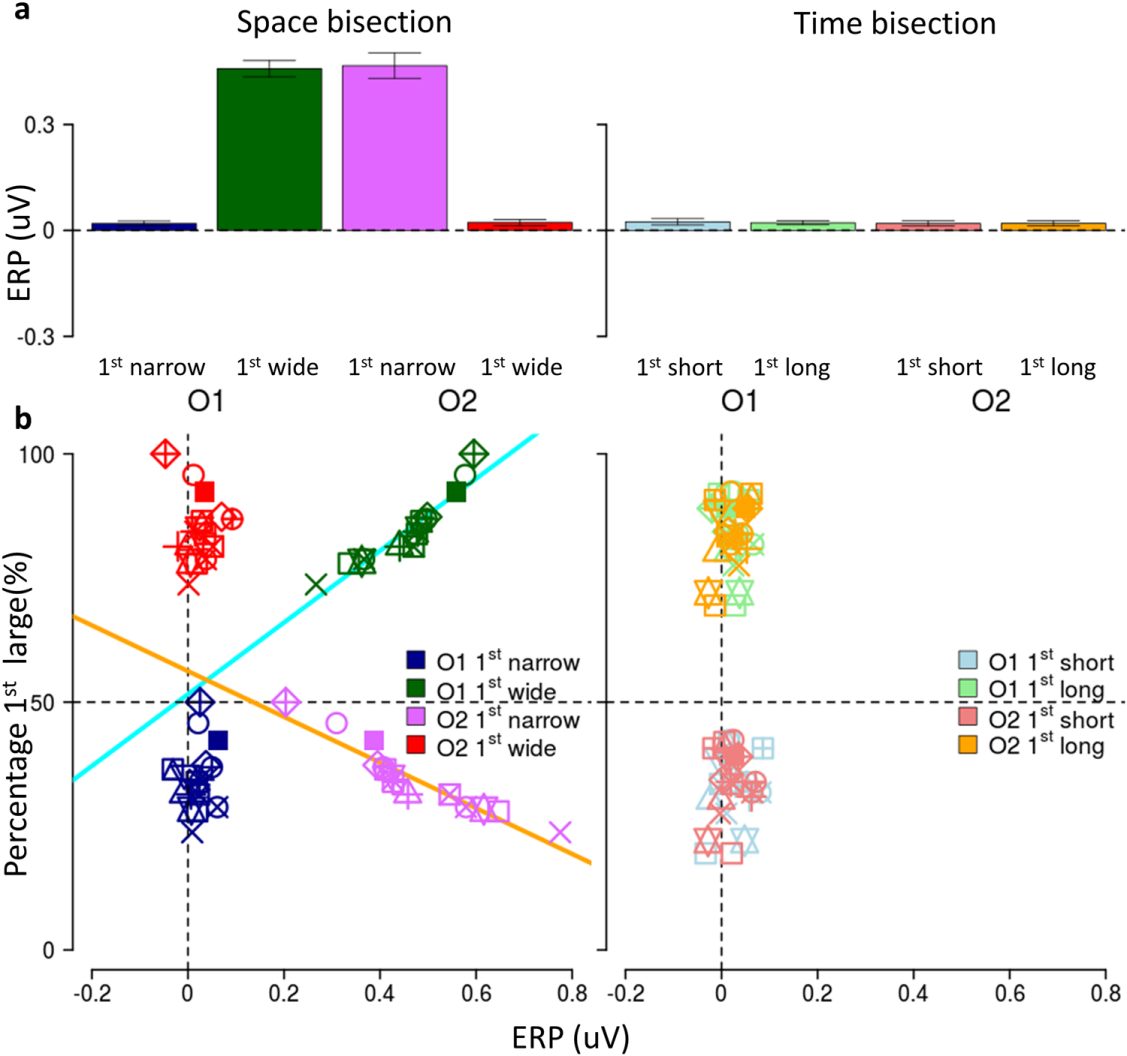
Relationship between early occipital response and position of second sound. **(a**) Average amplitude across subjects of early response, estimated as mean voltage in the time window between 50 and 90 ms after the sound. On the left, spatial bisection: average of trials in which S2 is presented in the left or in the right hemispace (mean ± SEM). On the right, temporal bisection: average trials with S2 presented with shorter or longer temporal separation from S1. (**b**) Correlation between the relative localization of the second sound with mean ERP amplitude. Individual mean ERP amplitude in the 50–90 ms time window plotted against the percentage of perceiving the first interval larger for spatial bisection task (left) and temporal bisection task (right) in O1 (blue and green) and O2 (red and pink). Each point corresponds to a single subject and the percentage was computed for the two separate spatial and temporal intervals. Cyan and orange lines represent significant correlation.

Figure 4a, showing the average of the voltage signal in the 50–90 ms time window, points out more clearly the difference in the acoustic response between the tasks. Early occipital response to the second sound was highly statistically significant during the space estimation task (Fig. 4a Left, t_15_ = 4.6, P = 0.0003), but not so significant for the time estimation task (Right, t_15_ = 1.14, P = 0.27).

The overall average early component amplitude (Fig. 4a) was similar for O1 and O2 electrodes, with similar gain modulation depending on the task. The lack of an occipital response to the second sound during the temporal bisection task was not due to a summation of opposite polarity waveform at the electrode. The early responses for the two separate spatial positions for the short and long temporal intervals were always equal in polarity and amplitude and overall small and just measurable (for short interval left 0.04 ± 0.06 µV, t_15_ = 0.67, P = 0.51, right 0.02 ± 0.05 µV, t_15_ = 0.4, P = 0.69; for long interval left 0.03 ± 0.06 µV, t_15_ = 0.50, P = 0.62, right 0.05 ± 0.07 µV t_15_ = 0.71, P = 0.49).

To probe whether the response modulation is related to the relative position, rather than to the physical position of the second sound, we correlated individual performance with individual ERP responses. As shown in Fig. 4b, the correlation between early occipital ERP component amplitude of O1 and O2 and individual subject performance was very strong and lateralized for the spatial task (for O1 and wide first interval R = 0.90 P = 0.000002, for O2 narrow first interval R = −0.89 P = 0.000004, while for O1 and wide first interval R = 0.07 P = 0.8 and for O2 and wide first interval R = −0.13 P = 0.63) but not for the temporal task (for O1 and long first interval R = 0.1 P = 0.71, for O2 and short first interval R = 0.13 P = 0.32, for O1 and short first interval R = −0.04 P = 0.88, for O2 and long first interval R = 0.05 P = 0.85), suggesting a pivotal role of the mental representation of auditory space in modulating early visual cortices.

We also observed a gain modulation of the later responses (P140) during the spatial task, selective again for the second sound, but not a lateralization effect, confirming the specific role of early visual cortex in observed early response (Fig. 3).

Given that both tasks were performed with equal precision (P correct: for space was 0.89 ± 0.10, for time is 0.83 ± 0.11, t_15_ = 0.65, P = 0.52) and similar reaction times (space = 0.94 ± 0.56 s, time = 0.96 ± 0.47 s, t_15_ = 0.30, P = 0.77), we can exclude that the selectivity of the response for the second sound reflected a difference in difficulty of the subject in performing the task.

**Figure 5:**
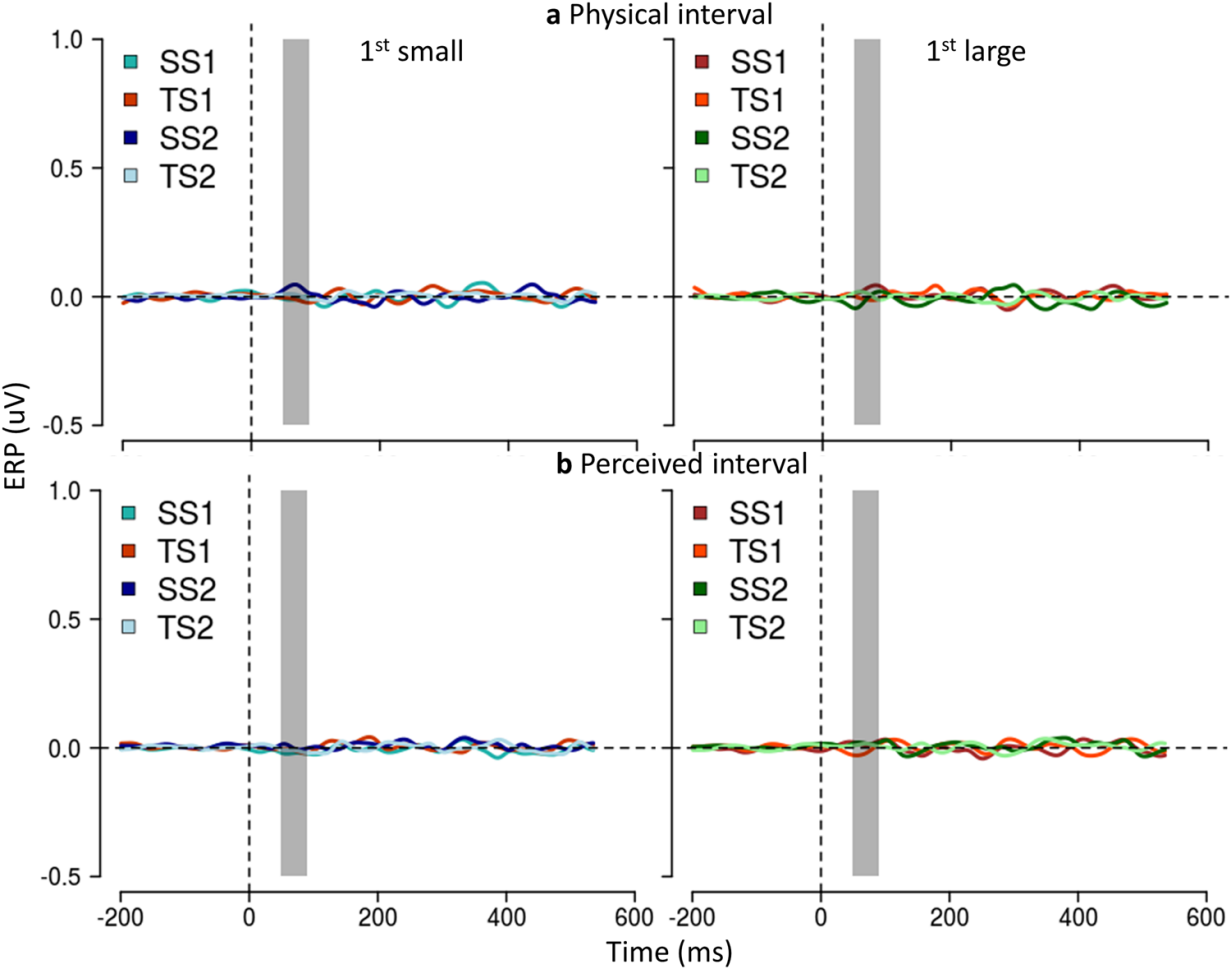
Amplitude of ocular movements is not modulated by relative position of the second sound. Mean amplitude of ocular movements is calculated as the difference between the left and the right EOG (mean ± SEM). On the left, average of trials in which the first interval was relative as smaller (for space bisection this corresponds to S2 relative on the left); on the right, trials in which first interval was relative as larger (for space bisection this corresponds to S2 relative on the right). Curves in each sub-plot represent the first and the second sound (S1, S2) for temporal (TS1, TS2) and spatial bisection task (SS1, SS2). t = 0 corresponds to the sound onset. No significant deflection emerged grouping by either physical (**a**) or by relative intervals (**b**).

We can also exclude that the effect originated from spurious eye-movement towards the apparent location of the sound. Average response of the eye deviation measured by EOG was equal and not significantly different from zero, both when grouping responses by physical (for the lowest P value t_15_ = 0.27, P = 0.81, Fig. 5a) or by relative (for the lowest P value, t_15_ = 0.33, P = 0.74, Fig. 5b) position of the second sound.

Furthermore, to clarify that the early occipital positivity and other later responses did not depend on the timing of the intermediate sound, we analyzed data for the spatial task separately for the short and long time interval (see Supplementary Figs 2–7). There was no interaction between the observed experimental modulations of the ERP responses and the timing (early/late) of the intermediate sound.

The results explained above show that the specificity of the gain modulation occurred only for the second sound, only for spatial bisection tasks and at early processing in the same time window of the visual C1 ERP component, see^37–39^, closely mimicking the contralateral activation of the occipital visual cortex^40^.

Whatever the mechanism, it is important to stress that the effect is specific to the occipital electrodes at early processing. Early responses in central (C1 and C2) or in other electrodes do not show the lateralization effect or the correlation with the behavioral responses (Figs 2 and 3).

### Source Level Analysis

To provide more evidence that the early positivity over the occipital scalp was actually involving generators in occipital areas, we performed comparisons at source level.

**Figure 6:**
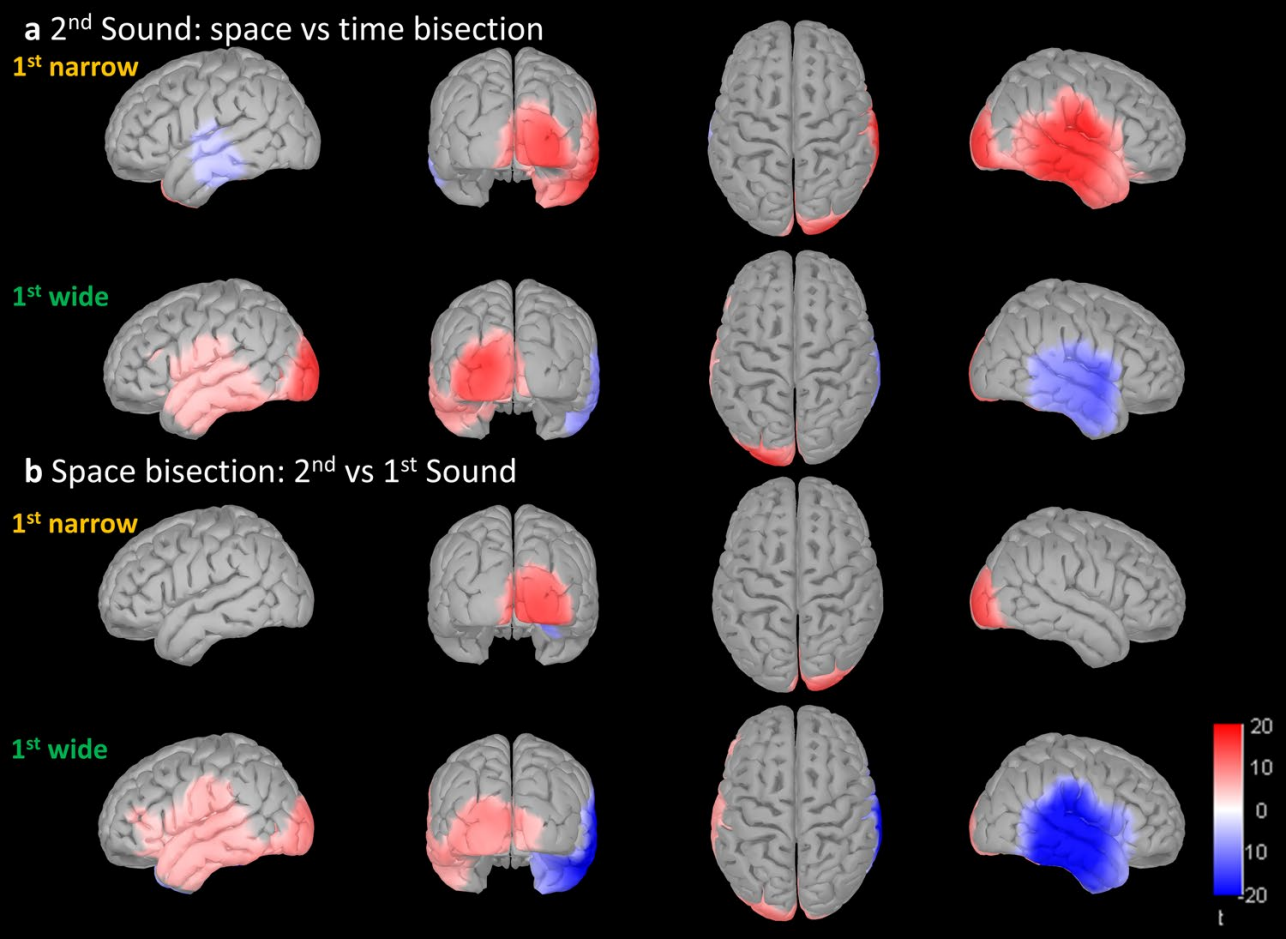
Second sound during space bisection elicits a specific early activation of contralateral visual cortex. Results of pairwise two tailed t-tests performed on average source activity in the (50–90 ms) time window. On each line, different views of the same comparison between cortical activations; from left to right: left, back, dorsal and right. Comparisons were performed (**a**) between space and time bisection tasks after second sound and (**b)** between the second and the first sound within spatial bisection task. Signed values of t are displayed: reddish colors indicate that space bisection (**a)** or second sound (**b**) produced a stronger cortical activation respectively than time bisection or first sound. Only t values corresponding to p < 0.0001 after FDR correction are displayed.

Considering the second sound, space bisection elicited a cortical response in the temporal region that was contralateral to the physical position of the sound, while time bisection produced a bilateral temporal activation. Moreover, space bisection produced a strong and specific occipital activation, which was contralateral to sound position. Therefore, compared with time bisection (Fig. 6a), space bisection elicited, after the second sound, a stronger early activation in the hemisphere contralateral to the physical sound; not only in temporal areas related to auditory perception, but also in occipital areas related to visual perception. However, as expected for bilateral activation, time bisection showed stronger activation in ipsilateral temporal areas.

On the other hand, considering early response during spatial bisection (Fig. 6b), the first sound activated the right temporal cortex, as expected given that the first sound was always presented on the left of the subjects. The second sound elicited a temporal activation which had a similar magnitude to the first sound and was contralateral to sound position. However, during the spatial bisection task, the second sound specifically activated the occipital cortex more than the first sound did. This result seems to confirm the specific role of the second sound as the starting point for building a metric in the space bisection task.

The second sound during the spatial bisection task also produced a specific activation of an extended dorsal network in the P140 time window (Supplementary Figure 9), as well as a contralateral response of the parieto-occipital cortex during the ACOP time window (Supplementary Figure 10).

## Discussion

Here we demonstrate for the first time in a purely auditory bisection task and in blindfolded naïve sighted subjects, the existence of an early occipital ERP component which is specifically elicited by the construction of a spatial metric.

Many studies, using a variety of techniques, have shown visual cortical activation to sounds in sighted individuals, by measuring the modulation of the visual response, manipulating the congruency between visual and acoustic signals^13, 19, 24, 41, 42^. Recent ERP studies have shown an auditory-evoked contralateral occipital activation (ACOP) in sighted individuals, which seems to respond to the experimental context^26^. However, ACOP only occurs in a late time window, between 250 and 400 ms after the sound^22, 23, 26, 27^. However, here we demonstrated a strong response to sound presented alone in the same early time window as a visual-evoked C1, suggesting that the signal originates at the level of early sensory cortices and that the same mechanisms of early analysis could be recruited when subjects have to solve an audio-visual multisensory task and when they have to localize a sound in space. In fact, the latency onset of the audio-visual interaction is reported to be coincident with the onset of the early component of the Visual Evoked Potentials, which is generated by the thalamic input and the first processing in the primary visual cortex^19, 24, 37–39, 43, 44^.

Interestingly, we observed this early response to sound in naïve sighted subjects. The component found here seems to have generators in the occipital area and not to be the same N48 previously described by Schroeder and colleagues whose generators would be in parietal areas^45^. N48 occurred after auditory stimuli presented alone, when randomly intermixed with visual stimuli presented alone and auditory and visual stimuli presented simultaneously. Moreover, the observed early occipital component mimics many characteristics of the C1 component generally characterizing responses of early striate visual cortex. Therefore, a possible interpretation our findings is that, while N48 is more involved in a multisensory integration process, an auditory response of the visual cortex exists in the same time window as the visual-evoked C1, which is specifically modulated by the construction of a spatial metric. More generally, we can speculate that cortical activation underlying C1 ERP component could play a fundamental role in the construction of metrics in the space domain, independently from the used sense. The recruitment of extrastriate visual cortices, including LOT, V5, hMT+ and EBA, is commonly reported in early blind and in trained sighted subjects^46^, as well as in naïve sighted subjects^22, 23, 47^. A recruitment of striate cortical areas in purely auditory tasks is commonly reported in blind subjects^46, 48–53^ but rarely in sighted subjects and only by fMRI^13^ or TMS techniques^17^.

The other important result is that the early occipital ERP component is selective for the spatial position of the sound. We observed a strong contralateral response with respect to the physical or the relative position of the sound. A similar pattern has been reported for the spatial position of visual stimuli, suggesting that the audio response could be mediated by the same map. The spatial distance of the sound target from midline was very close to spatial discrimination threshold for most subjects and sub-threshold for others, who performed near chance level. Crucially, when we correlated the percentage of trials in which subjects judged the first interval as larger with the amplitude of early occipital ERP component, we observed a strong correlation only for the spatial bisection, only for the second sound and only in occipital electrodes. This indicates that the spatial selectivity of early occipital component is linked to the relative spatial localization and not only to the physical position. Two alternative interpretations of this result are possible: the auditory relative localization may be elaborated before interaction with the visual system; or alternatively, the interaction of the auditory signals with the visual occipital cortex could define the relative localization of the sound. It is also possible that the decision regarding the target localization of the subject could be the origin of the effect, as has been previously demonstrated for visual stimuli or decisions, see^54^. However, this is not consistent with the short latency of the auditory response, thus strengthening the idea that the response reflects an early sensory-sensory interaction.

When the subjects performed a temporal discrimination task we never observed any strong early occipital response. This, in principle, could result from the annulling of the positive and negative polarity components that sum out of phases. However, we also observed similar results when separately considering physical position of the second sound, therefore excluding this mere summation effect. Previous fMRI studies^15^ have shown that auditory temporal expectations modulate activity in the visual cortex. However, our data regarding temporal bisection did show a stronger early occipital activation after the second than after the first sound. This difference could indicate that the construction of temporal metrics relies only partially on expectation, or that expectation could involve later time windows. On the other hand, during spatial bisection, early occipital activation increased from the first to the second sound: we cannot exclude, therefore, that one of the processes involved in the construction of auditory spatial metrics could be temporal expectation. We can also exclude that our effect merely originated from attention to the left or right positions of the sound. We never observed a modulation in central electrodes, which are those that usually show strong attentional responses to sounds^55, 56^. Similarly, mere attention to space cannot explain the experimental pattern and modulation of occipital C1 ERP component.^37, 57–59^ Contrarily, it seems that spatial attention mostly affected the N1 for central electrodes^55, 56^.

Our results are also consistent with the numerous multisensory audiovisual studies, where a clear retinotopic interaction has been demonstrated^13, 14, 17, 42^ and with the animal-model anatomical evidence for multisensory interactions at early stages of cortical processing. There are direct feedback connections from primary auditory and multisensory areas to V1 and V2 in macaque^11, 12, 21^. Space perception is a difficult task. The brain uses several different strategies to solve it, from the building of neural selectivity linked to the external world, independently from the position of the subject, to a dynamic and predictive update of all senses’ frames of reference before a shift of sensory position^33, 60^. The auditory spatial bisection task often requires the transformation of a head-centered auditory representation into an allocentric representation. Recent data from our laboratory have also demonstrated that vision plays a leading role in acoustic space perception e.g.^61–64^: a similar auditory spatial bisection task used here is severely impaired in the congenitally blind who have never experienced a visual sense of space^62^.This, together with the present demonstration of a selectivity of the response of the visual cortex to sound, indicates that the visual occipital cortex may play a key role in the transformation from the head-centered to allocentric coordinate system. In addition, our data show that this transformation is functional every time we have to locate a sound source and not only in pathological subjects or during plastic remodeling of the visual cortex. We can speculate that coordinate transformation could be mediated by pathways involving the superior colliculus, as previously proposed^62, 65, 66^, while we can exclude that our data reflect an indirect auditory activation mediated by the acoustic thalamus^20^, given that we found no modulation or spatial selectivity for central electrodes that record auditory cortical processing. The same applies also during a task that does not require the building of spatial map, such as during the temporal bisection task.

Admittedly, even with source analysis, we cannot provide a precise indication of the location of the cortical areas which generate our early occipital component or of a real retinotopic pattern. Specifically, generators of the observed component generically involve the visual cortex, while those of visual-evoked C1 specifically involve the primary visual cortex, therefore we cannot sustain a direct association between the two ERP components. Nonetheless, we can claim that a task based on space metric – therefore, mostly calibrated by vision^62^ - produced a specific response (1) in the same time window as visual C1 (50–90 ms) which is also crucial for earliest stages of multisensory integration; (2) in the same scalp areas, i.e. in occipital and, most importantly, not in central or in other more anterior electrodes and with generators involving visual cortex; (3) not after the first, but after the second sound, which is the starting point for building a metric; (4) contralateral to the second sound and strictly correlated to the relative sound position, as would be expected for a visual stimulus. Therefore our data seem to provide robust cues of a pivotal role of the primary visual cortex in the perception of space, whatever the sensory modality of the stimulus. The acoustic recruitment of the visual brain may be necessary to build a spatial metric of the environment with the high resolution and flexibility that only the visual brain is able to implement.

## Methods

### Participants

16 healthy blindfolded sighted adult subjects, 11 females and 5 males with a mean age of 42 years and standard deviation of 16 years were recruited to participate in this study. All participants reported normal hearing. All participants gave written informed consent before starting the test. The study was approved by the ethics committee of the local health service (Comitato etico, ASL 3, Genoa) and was conducted in line with the Declaration of Helsinki.

### Stimuli

Three short sounds (namely S1, S2, S3; 500 Hz, 75 ms duration, 60 dB SPL at the subject position) were delivered at three different spatial positions (Fig. 1a **)** and times (Fig. 1b) using free-field speakers placed in the lower visual hemifield.

The first (S1) and third sounds (S3) were always delivered at −25 and +25 degrees respectively; the temporal separation was fixed at 1.5 seconds. The second sound (S2) could come in space from either −4.5 ° or 4.5 ° from trial to trial (Fig. 1a), and, independently, in time either at −250ms or +250ms ms with respect to the middle of the sound sequence (Fig. 1b). These values correspond to approximately 75% of correct answers for spatial and temporal bisection thresholds evaluated in a preliminary session with 5 subjects. Inter-trial interval was 1250 + /− 250 ms.

### Procedure

Subjects performed two bisection tasks, one spatial and the other temporal, in two separate blocks. The order of the blocks was randomized between subjects. It is important to note that the stimuli were identical in the two blocks. In each block, subjects evaluated whether the first interval (between S1 and S2) was smaller or larger than the second interval (between S2 and S3) in space (referred to as “narrow” and “wide” respectively, see Fig. 1a) or in time (refereed to as “short” and “long” respectively, see Fig. 1b).

For the sake of clarity we refer to “small” and “large” when we refer to the intervals between the sounds irrespective of space and time while we refer to “long” and “short” in relation to time and “narrow” and “wide” in relation to space.

The temporal separation between sounds was large enough to allow a complete decay of the ERP response.

To avoid possible spurious neural responses, subjects were asked to answer immediately after the third sound. Therefore, we measured the subject reaction times (RT), as the time between the third sound and the button press, and the subject performance, i.e. the percentage of responses ‘larger’ for the first interval. For statistical analysis, subject accuracy (% correct) was Z-transformed, given that spatial and temporal separation obey the Weber law.

### EEG data collection and pre-processing

EEG was recorded with 64 active electrodes (Biosemi Active 2 EEG System). Preamplifiers in each electrode were used to reduce induced noise between the electrode and the amplification/digitization system electrode and the amplification/digitization system (BioSemi ActiveTwo, BioSemi B.V. Amsterdam), allowing high electrode impedances. Electrode offsets were kept below 35 mV. A first-order analog anti-aliasing filter with a half-power cutoff at 3.6 kHz was applied (see www.biosemi.com). The data were sampled at 512 Hz (2048 Hz with a decimation factor of 1/4) with a bandwidth of DC to 134 Hz, using a fifth order digital sinc filter. Each active electrode was measured online with respect to a common mode sense (CMS) active electrode producing a monopolar (non-differential) channel.

Two additional electrodes were positioned on the external canthi (the bone at the side of the eye) for EOG recording. Particular care was taken to avoid the blindfold touching any electrodes. Eye movements produce a moving dipole source, generating positive and negative peaks when differentiating the two EOG electrodes, representing eye movements toward the right and left respectively. Trials showing horizontal ocular movements were discarded. In addition, to exclude possible spurious effects due to ocular movements, we calculated the difference between left and right EOG and averaged the signal in synchrony with the first or second sound presentation. We then compared amplitude of ocular movements between conditions, and no significant modulation due to the physical or relative spatial position of the second sound was measured.

EEG was filtered between 0.1 and 100 Hz. Next, transient, high-amplitude artifacts from stereotypical (e.g., eye blinks) and non-stereotypical (e.g., movement, muscle bursts) were removed using an automated artifact rejection method termed Artifact Subspace Reconstruction (ASR)^67^ which is available as a plug-in for EEGLAB software^68^. ASR uses a sliding window technique whereby each window of EEG data is decomposed via principal component analysis so it can be compared statistically with data from a clean baseline EEG recording, collected here as 1 min of EEG recorded during quiet standing. Within each sliding window the ASR algorithm identifies principal subspaces which significantly deviate from the baseline EEG and then reconstructs these subspaces using a mixing matrix computed from the baseline EEG recording. In this study, we used a sliding window of 500 ms and a threshold of 3 standard deviations to identify corrupted subspaces.

Furthermore, channels were removed if they were less correlated than 0.85 to an estimate based on other channels, or if they had more line noise relative to its signal than 4 standard deviations based on the total channel population.

Time windows were removed when, after the application of the previously described criteria, the fraction of contaminated channels exceeded the threshold of 0.25. Other parameters were kept as their default.

EEG data were further cleaned using independent component analysis^68^.

Specifically, to select artefactual components based on quantitative criteria, we used two EEGLAB toolboxes, namely SASICA^69^ and IC_MARC^70^, keeping all parameters as their default and following, for component rejection, the criteria reported in the corresponding validation papers, mainly based on abnormal topographies and/or spectra. For the sake of transparency, in Supplementary Figures 11–14 we reported ERPs obtained from pre-cleaned data, showing that electrophysiological modulations elicited by the task are still present, even if less evident due to the worse SNR.

Data were then referenced to the average of the left and right mastoids (TP7, TP8 electrodes).

### Sensor Level Analysis

Here we were specifically interested in verifying the hypothesis that only during spatial bisection the second sound, starting to elicit the development of a spatial metric, could produce a contralateral activation, which would mimic what is observed in visual tasks. Therefore, in this analysis we compared the neural response to the second sound with that of the first sound, taken as control, and we excluded the third sound, which could involve more complex mechanisms related to metric definition.

EEG data were averaged in synchrony with the first or second sound presentations to obtain the ERP, considering as baseline for both sounds a period of 200 ms before the first sound, therefore not affected by possible anticipatory effects.

For each subject, we presented 60 trials for each block and condition, and we requested a minimum of 40 trials for each ERP for the 2 spatial and 2 temporal conditions, after artefact removals.

In addition, each block presented 15 catch trials, with identical space and time intervals, which were not considered for statistical analyses of performances and ERPs. For each ERP, the total number of trials was equal to 855, around 55 per subject.

Both correct and incorrect trials were analyzed for two reasons: firstly, to increase statistical power; secondly, to investigate the relationship of the cortical activation with the response reflecting perceived extension of the first interval, independently from the matching with the physical extension.

In this study we considered electrodes linked to visual (O1, O2 in Occipital areas, see Fig. 2a) and auditory processing (C1, C2 in Central areas, see Fig. 2b), which could reveal a possible contralateral response with respect to the sound presented. A time window between 50 and 90 ms after the sound was defined by computing the grand average ERP (i.e. merging all conditions), therefore avoiding possible biases^71^, and resulted in agreement with previous studies evaluating the classical C1 ERP component observed in visual tasks^37, 57–59^. Notably, the selected electrodes were those showing the strongest response – considering the grand average, merging all conditions - in the selected time window.

Mean ERP amplitude was computed by averaging the voltage in the C1 ERP component time windows (50–90 ms, see Fig. 3).

We performed statistical comparisons using analysis of variance (ANOVA) considering the following as factors: AREA (Central, Occipital), HEMISPHERE (Left, Right), FIRST INTERVAL EXTENSION (Small, Large), DOMAIN INVOLVED IN BISECTION TASK (Space, Time) and SOUND (First, Second). Post-hoc comparisons were performed with paired two-tailed t-tests. Probabilities were retained as significant when lower than 0.05 after Bonferroni correction of 8 bisection task (spatial and temporal), Sound (S1 and S2) and Electrode (O1, O2, C1, C2).

The association between ERP response and subject performance was investigated using linear regression of individual mean ERP amplitude in the 50–90 ms time window against the percentage of trials in which the subject perceived the first interval as larger.

### Source Level Analysis

In order to reconstruct the cortical generators of the ERP components affected by the experimental factors, we employed a distributed sources analysis using the Brainstorm software^72^. Data were re-referenced to the common average^72^. Then, cortical current source distribution within the brain was represented through 15,002 elementary dipoles obtained by sampling a tessellated cortical mesh template surface derived from the standard 1 mm resolution brain of the Montreal Neurological Institute (non-linear average of 152 subjects, processed with FreeSurfer 5.3 ICBM152^73^). Since the individual MRIs were not available, and thus the dipole orientations derived from the template could not in any way approximate the actual brain geometry, dipole orientations were not fixed normal to the cortex surface but were let free to assume whichever (unconstrained) orientation. The EEG forward modeling of volume currents was completed with a three-layer (head, outer and inner skull) symmetric boundary element model (BEM) generated with OpenMEEG^74^. A diagonal noise covariance matrix was computed for each participant, using the pre-stimulus interval to estimate the sensor variance. The intensities of sources were estimated through a sLORETA approach^75^. This technique has been shown to be robust to noise in recorded data and head model approximations with fair spatial resolution, and the depth weighting used in this approach alleviates the natural bias of basic minimum norm estimation approaches toward superficial currents. Brainstorm’s default parameter settings have been used for both source reconstruction and BEM creation.

Source activation was averaged for each subject and condition within selected time windows; subsequently, the norm of the vectorial sum of the three orientations at each vertex was calculated. Finally, pairwise comparisons were investigated with paired t-test and results were corrected for multiple comparisons FDR method^76^, using p = 0.0001 as threshold. To probe the specificity of the activation after the second sound in the space bisection task, on one side we compared space with time bisection after the second sound; on the other side we compared the second with the first sound during the space bisection task.

## Electronic supplementary material

Supplementary Information
