# Supplementary material for: Spatial localization of sound elicits early responses from occipital visual cortex in humans
Source: Sci Rep. 2017 Sep 5;7:10415. doi: 10.1038/s41598-017-09142-z (PMC5585168; doi:10.1038/s41598-017-09142-z)
Supplement: Supplementary file 1 — Supplementary Information [file 41598_2017_9142_MOESM1_ESM.doc]

# Supplementary information

# Spatial localization of sound elicits early responses from occipital visual cortex in humans

Claudio Campus^1,2^, Giulio Sandini^2^, Maria Concetta Morrone^3,4^, Monica Gori^1,2^*.

# Sensor level analysis

## Grand average ERP

**
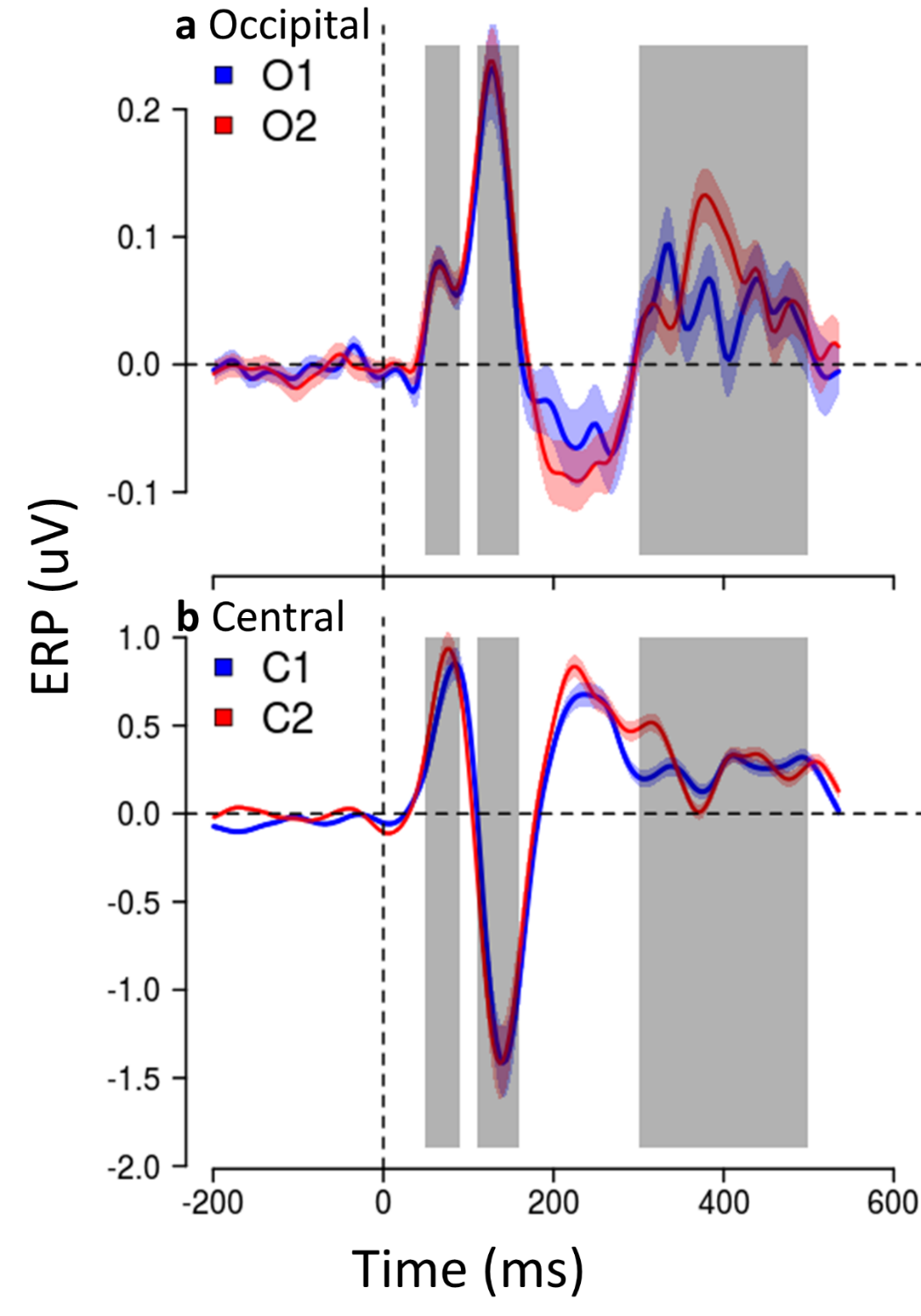
**

**Supplementary Figure 1**. Grand average ERP obtained by merging all experimental conditions across subjects. Waveforms averaged over the first and second sound. ERP (mean ± SEM) highlighted (see dashed rectangles) several responses modulated by experimental manipulation: an early response between 50 and 90 ms (early occipital positivity), a second response between 110 and 160 ms (P140) and a late response between 300 and 500 ms (ACOP) in Occipital (**a**) and Central (**b**) electrodes. t = 0 is sound onset.

## Responses splitted by time interval


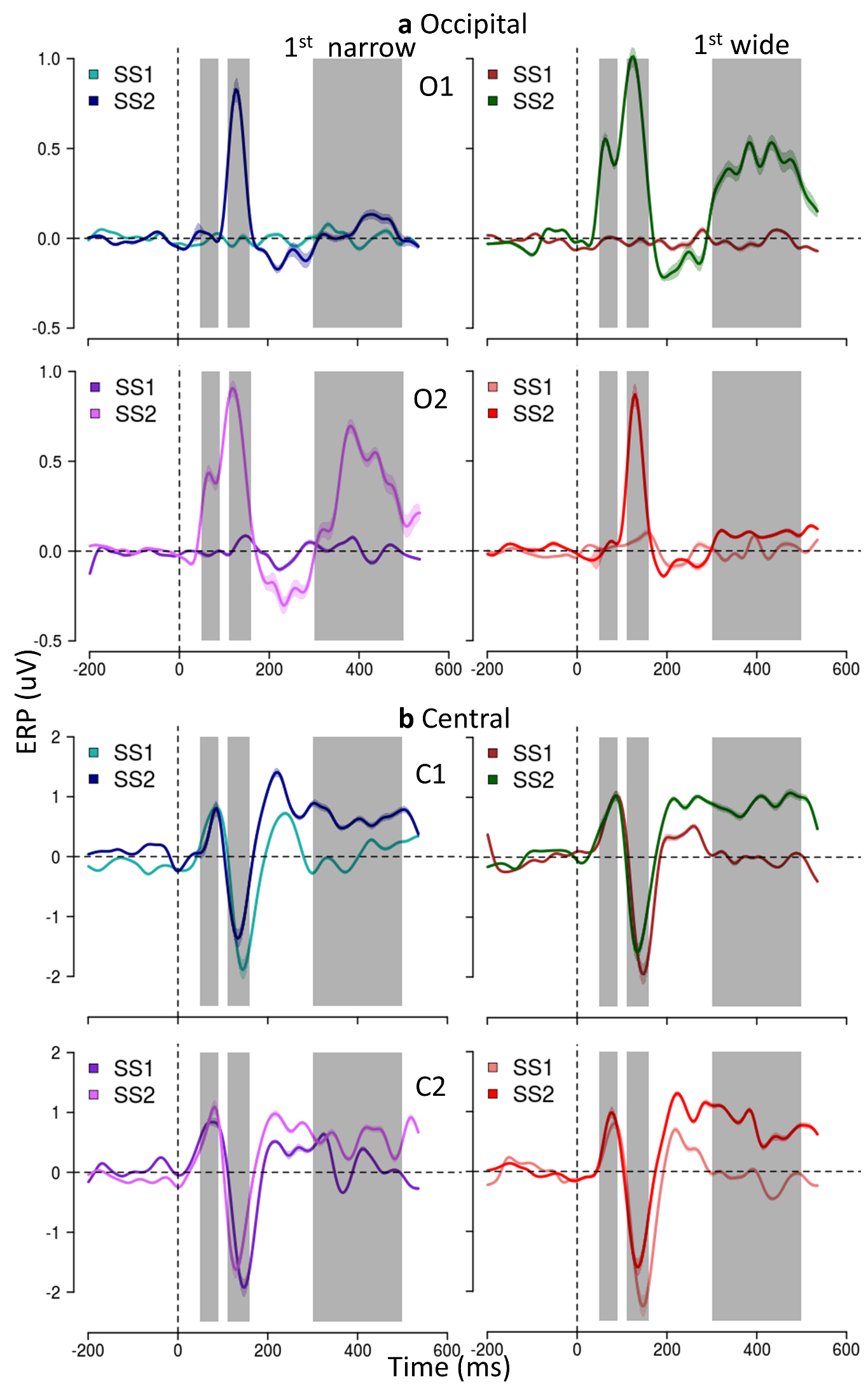


**Supplementary Figure 2.**  ERP for short first time interval. **a** Occipital areas. ERP (mean ± SEM) in O1, first raw, and in O2, second raw, averaged across subjects. On the left, average of trials in which S2 is presented in the left hemispace (spatial bisection); on the right, trials in which S2 is presented in the right hemispace. Differently colored curves represent ERP responses to the first and the second sound for spatial bisection task (SS1, SS2). t = 0 is sound onset. Shaded areas delimits early response between 50 and 90 ms (early occipital positivity), a second response between 110 and 160 ms (P140) and a late response between 300 and 500 ms (ACOP). **b** ERP in C1, first line, and in C2, second line (mean ± SEM).

**
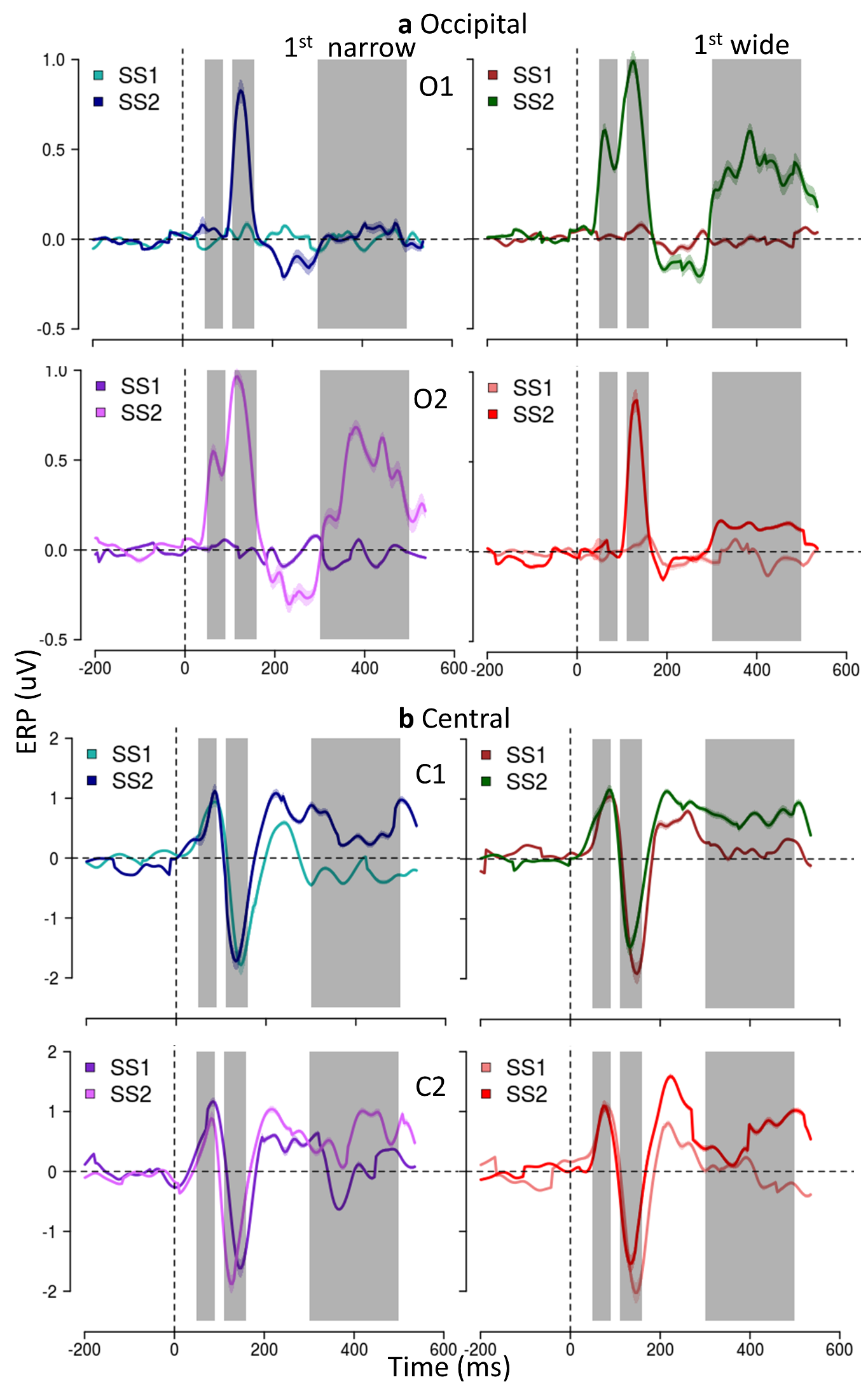
Supplementary Figure 3.** ERP for long first time interval. **a** Occipital areas. ERP (mean ± SEM) in O1, first raw, and in O2, second raw, averaged across subjects. On the left, average of trials in which S2 is presented in the left hemispace (spatial bisection); on the right, trials in which S2 is presented in the right hemispace. Differently colored curves represent ERP responses to the first and the second sound for spatial bisection task (SS1, SS2). t = 0 is sound onset. Shaded areas delimits early response between 50 and 90 ms (early occipital positivity), a second response between 110 and 160 ms (P140) and a late response between 300 and 500 ms (ACOP). **b** ERP in C1, first line, and in C2, second line (mean ± SEM).

## Early occipital positivity


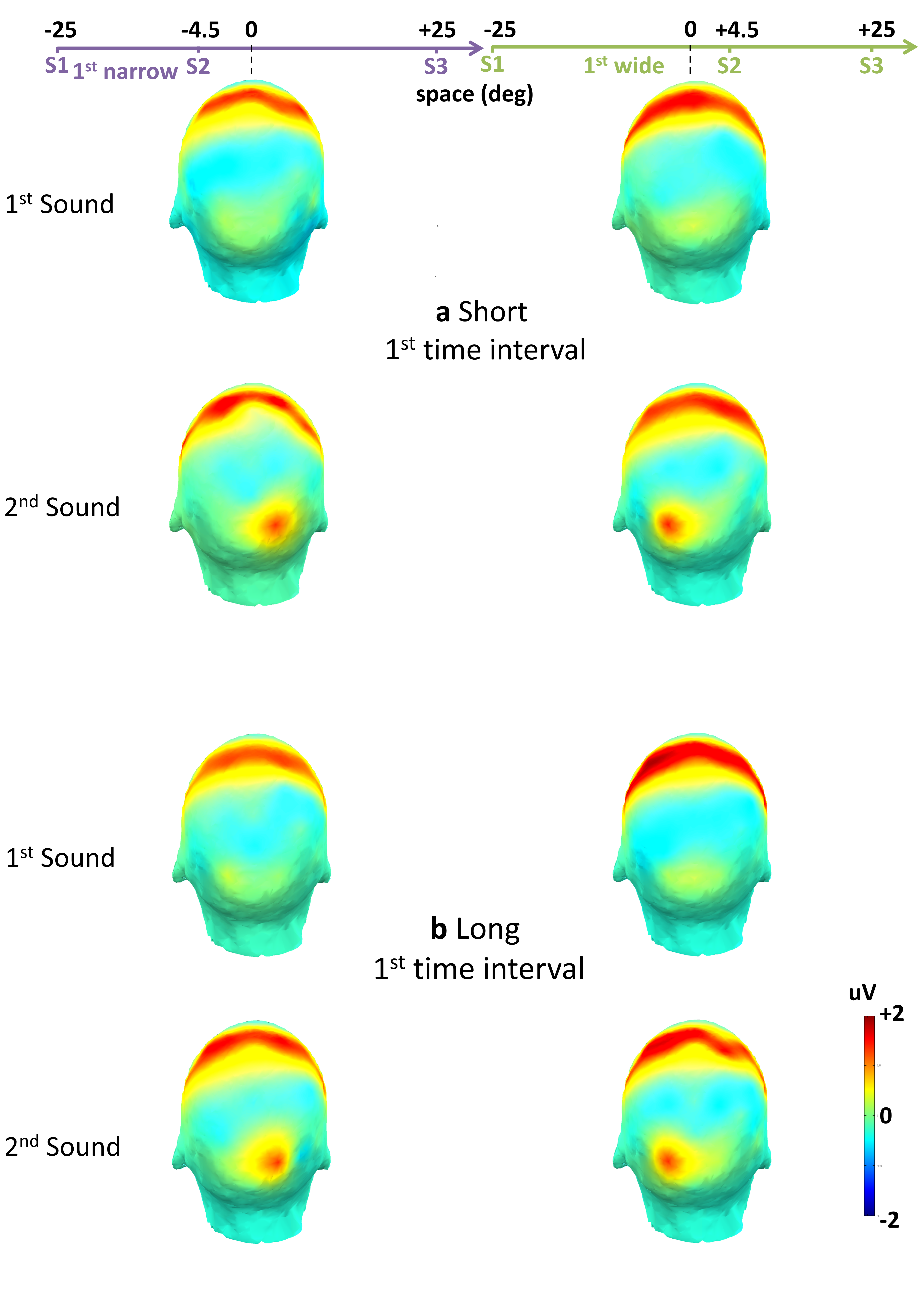


**Supplementary Figure 4.** ERP scalp map in the (50-90 ms) time window averaged across subjects during space bisection task. (**a)** Short and (**b)** long time interval showed similar patterns. For both durations of the first time interval, two strong positivities emerged. One, involving central areas, was not modulated by experimental manipulation. The other, involving parieto-occipital areas, showed a specific contralaterality when a narrow (Left) or a wide (Right) first interval respectively corresponded to a second sound in the left or in the right hemifield. This pattern was absent after the first sound.

## P140


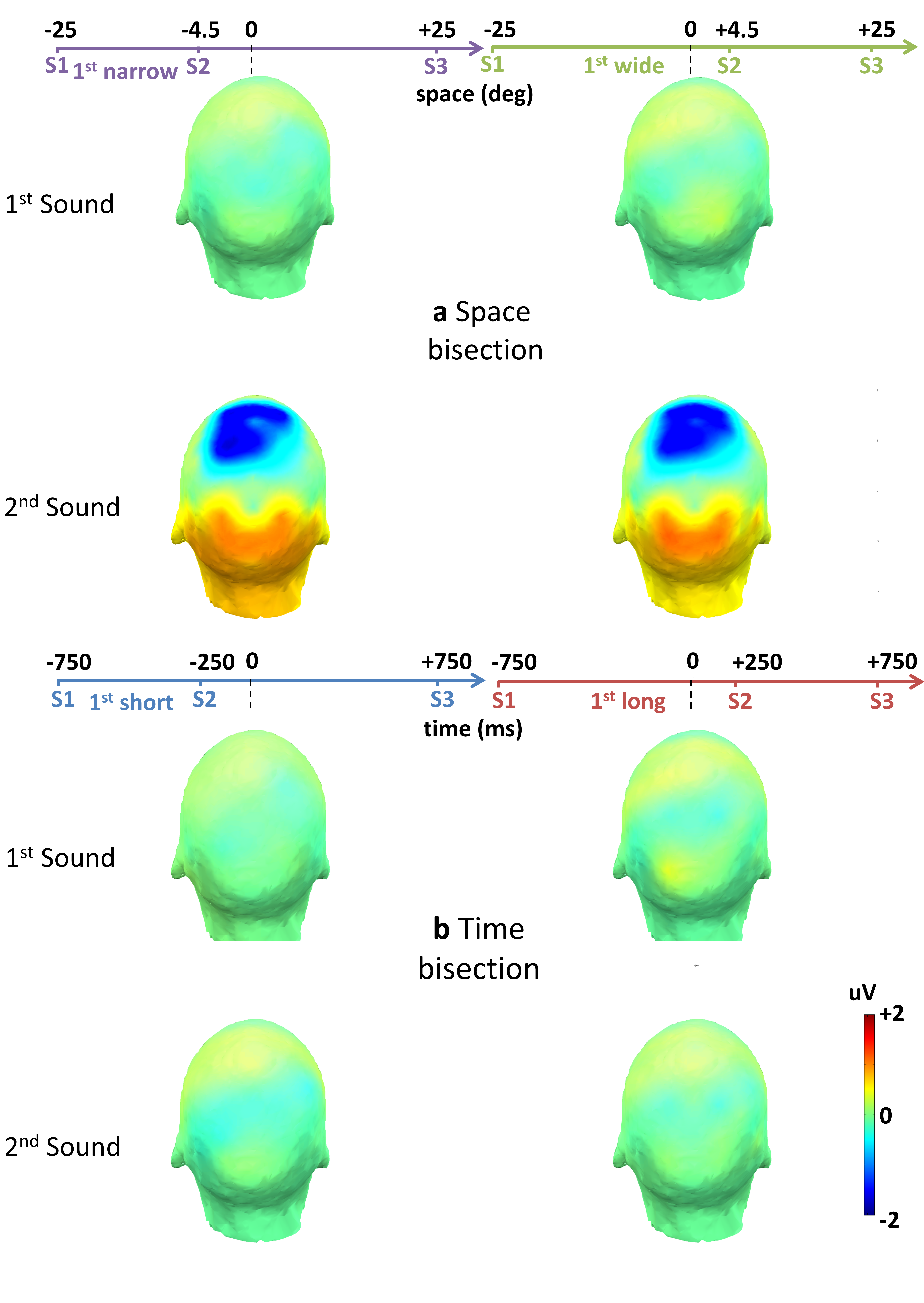


**Supplementary Figure 5.** ERP scalp map in the (110 -160 ms) time window averaged across subjects. During space bisection (**a**) the second sound produced a positivity in in parieto-occipital areas, while a negativity in front-central areas. Both positivity and negativity were not modulated by sound position. During time bisection task (**b**), a similar response was missing, as well as after the first sound for both bisection tasks.

**
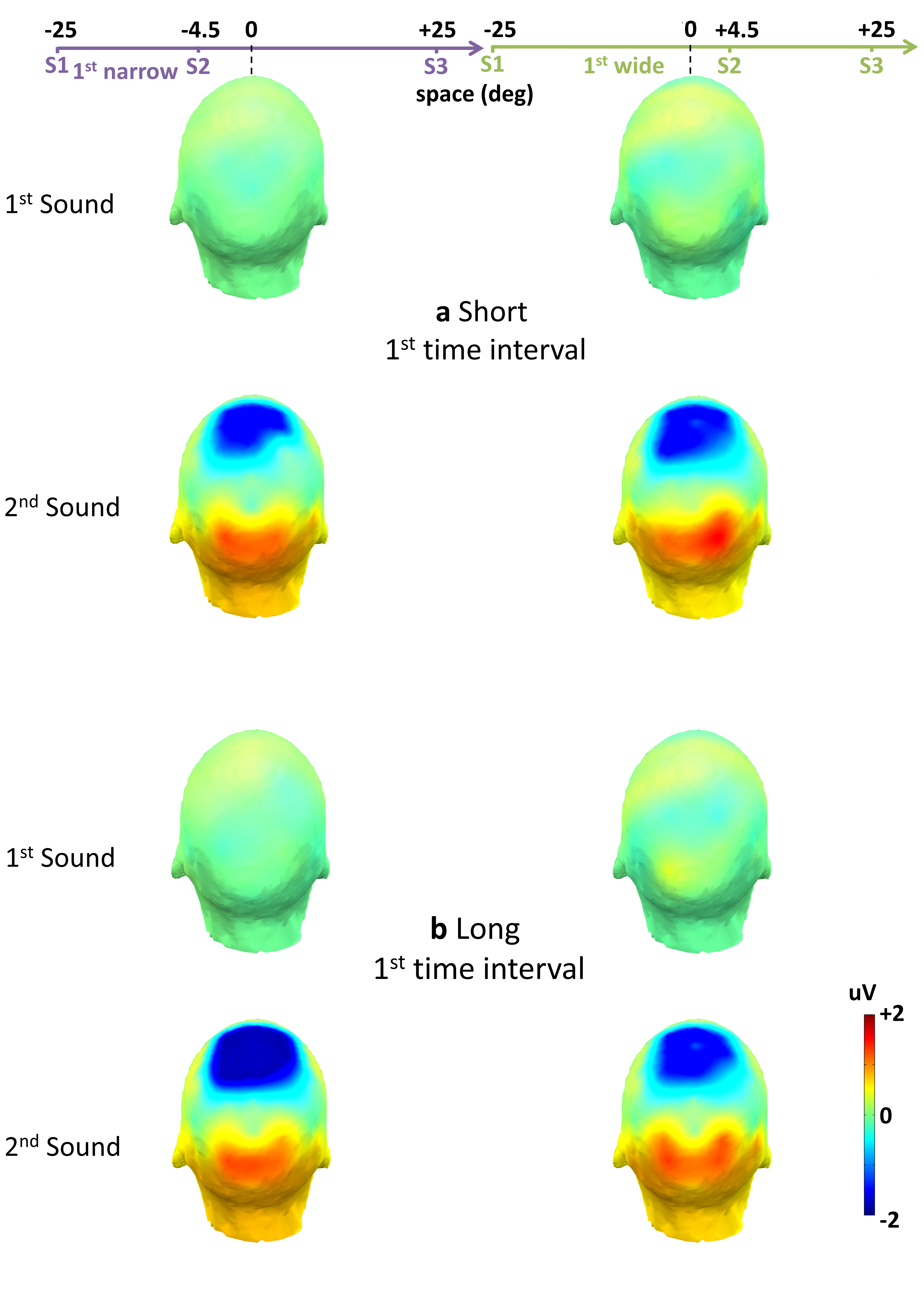
Supplementary Figure 6.** ERP scalp map in the (110-160 ms) time window averaged across subjects during space bisection. For both (**a)** Short and (**b)** long time interval a similar pattern was observed. During space bisection the second sound produced a positivity in in parieto-occipital areas, while a negativity in front-central areas. Both positivity and negativity were not modulated by sound position. A similar response was missing after the first sound.

## ACOP


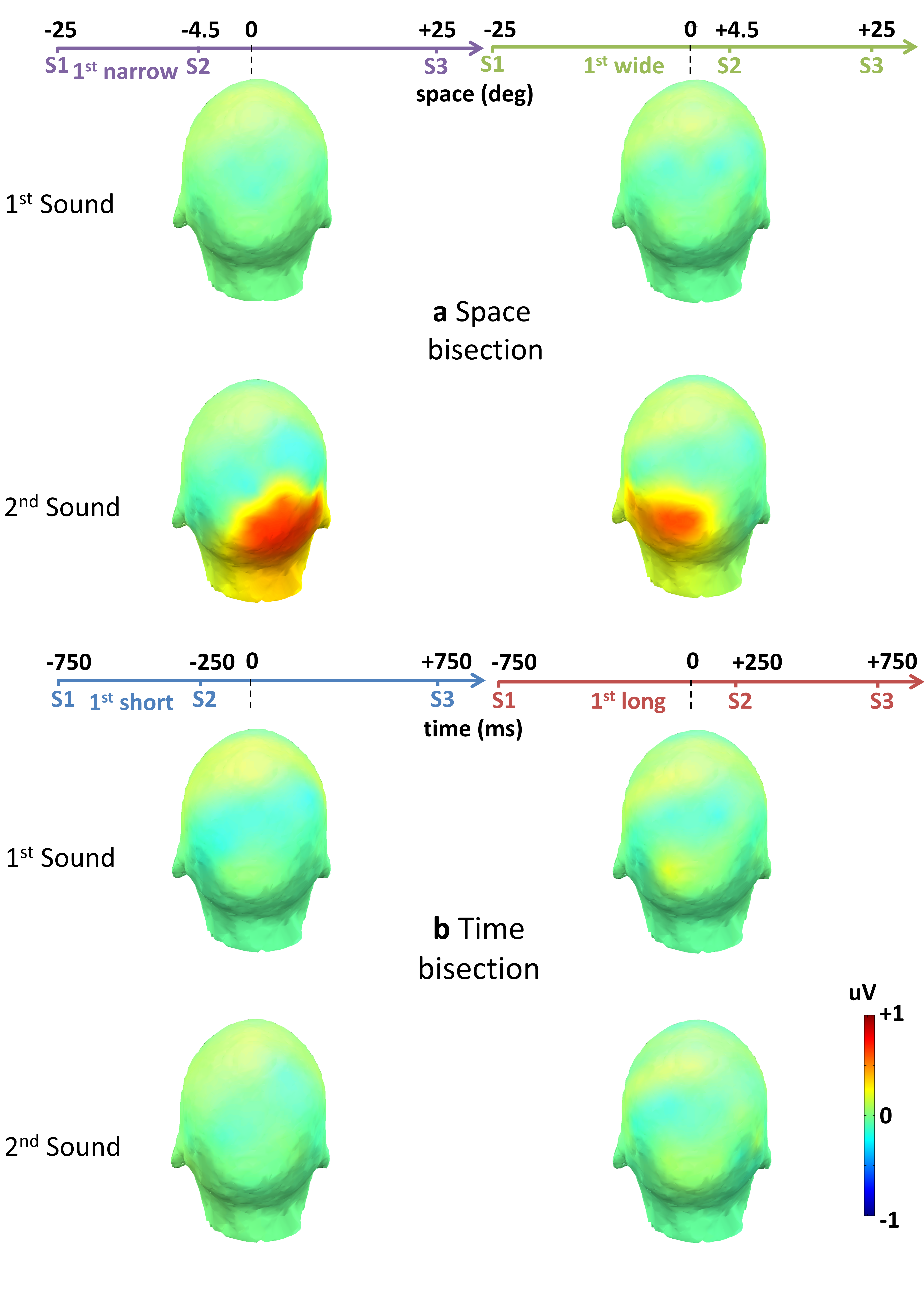


**Supplementary Figure 7.** ERP scalp map in the (300-500 ms) time window averaged across subjects. During space bisection (**a**) the second sound produced a positivity in in parieto-occipital areas contralateral to sound position, which was absent during temporal bisection (**b**) or, for both bisection tasks, after the first sound.

**
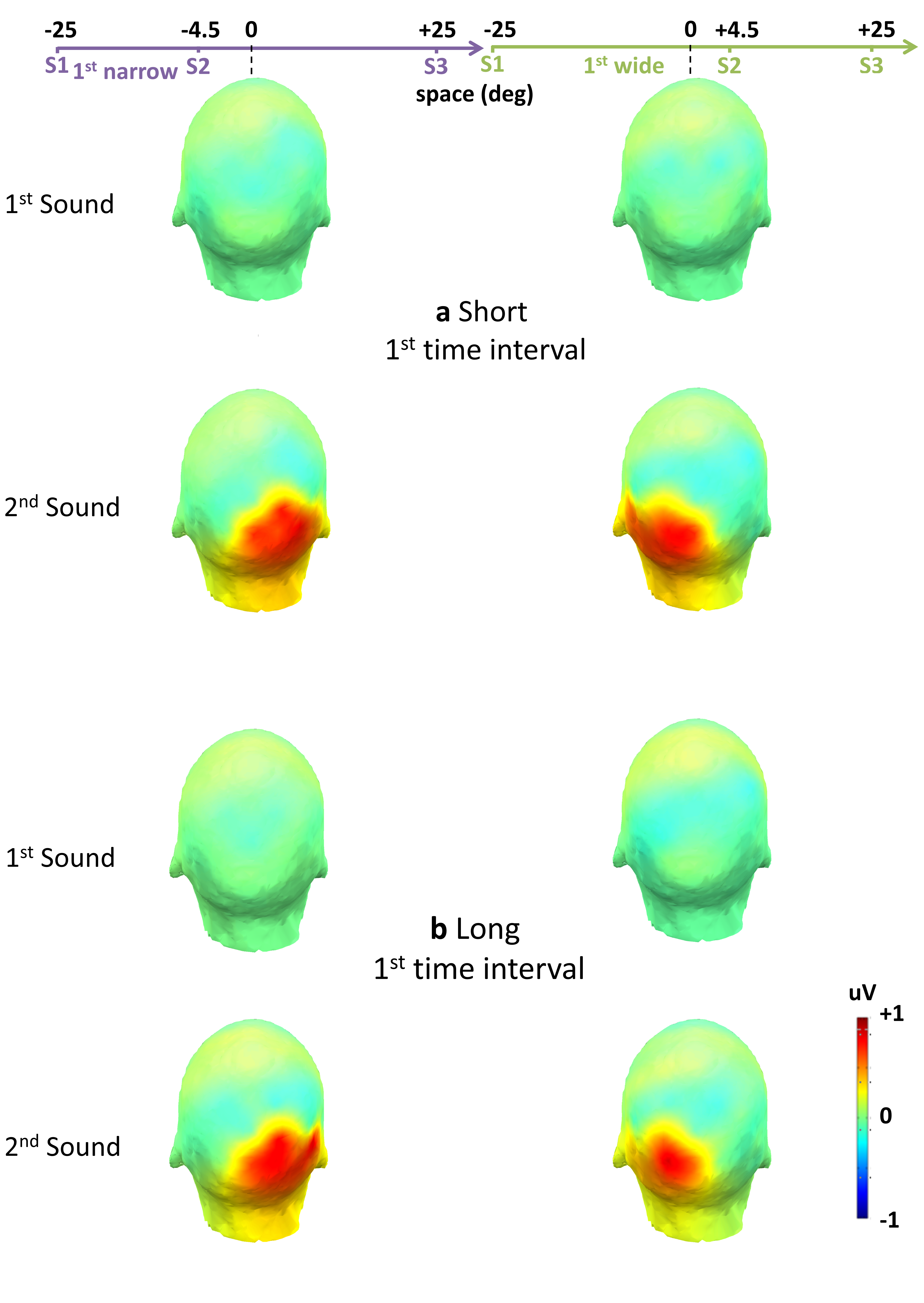
Supplementary Figure 8.** ERP scalp map in the (300-500 ms) time window averaged across subjects during space bisection task. (**a)** Short and (**b)** long time interval. For both durations of the first time interval, the second sound produced a similar positivity in in parieto-occipital areas contralateral to sound position, which was absent after the first sound.

# Source level analysis


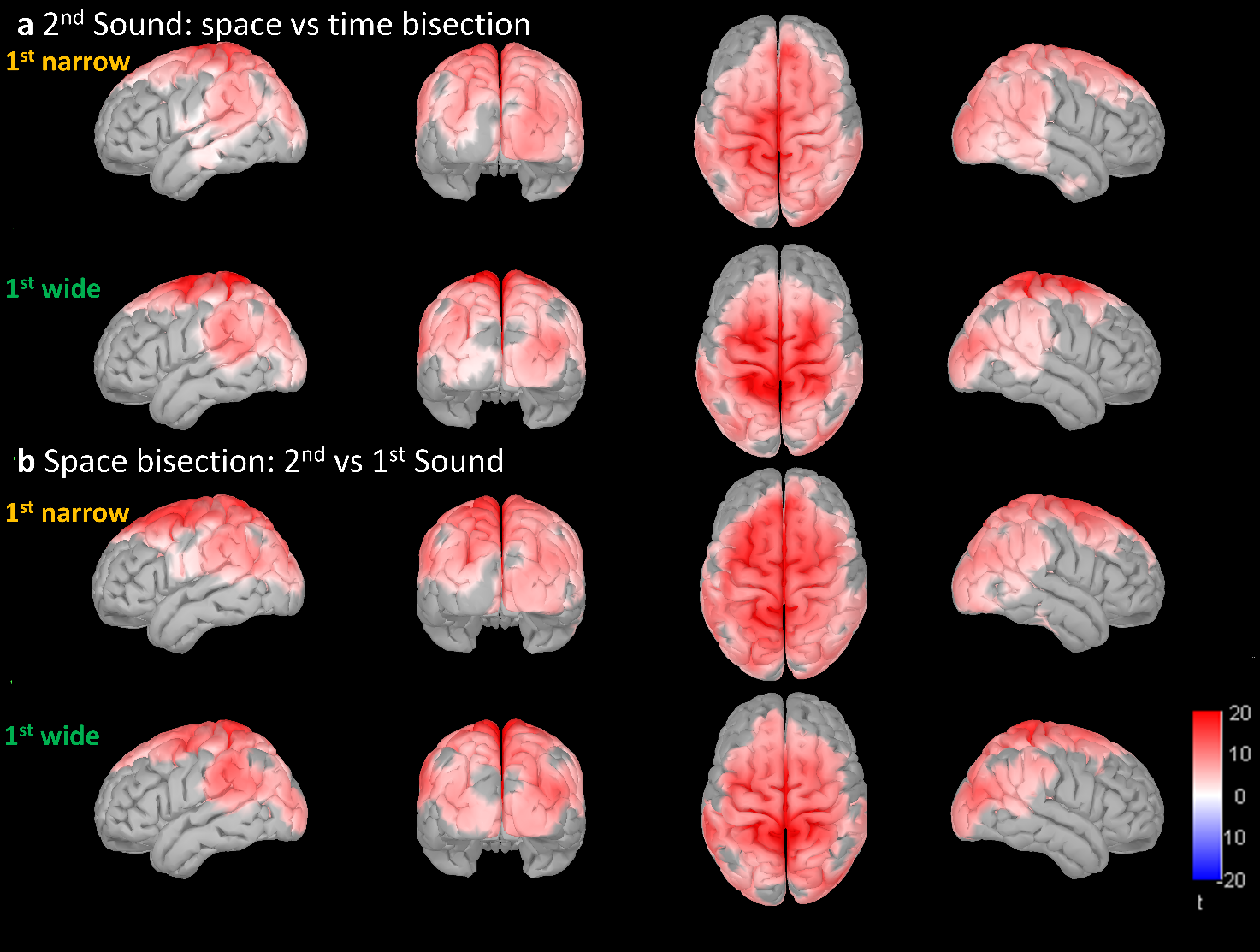


**Supplementary Figure 9**. Second sound during space bisection elicits a specific activation of an extended dorsal network. On each line, different views of the same comparison between cortical activations; from left to right: left, back, dorsal and right. Results of pairwise two tailed t-tests performed on average source activity in the (110-160 ms) time window. Comparisons were performed (**a**) between space and time bisection tasks after second sound and (**b)** between the second and the first sound within spatial bisection task. Signed values of t are displayed: reddish colors indicate that space bisection (**a)** or second sound (**b**) produced a stronger cortical activation respectively than time bisection or first sound. Only t values corresponding to p<0.0001 after FDR correction are displayed.


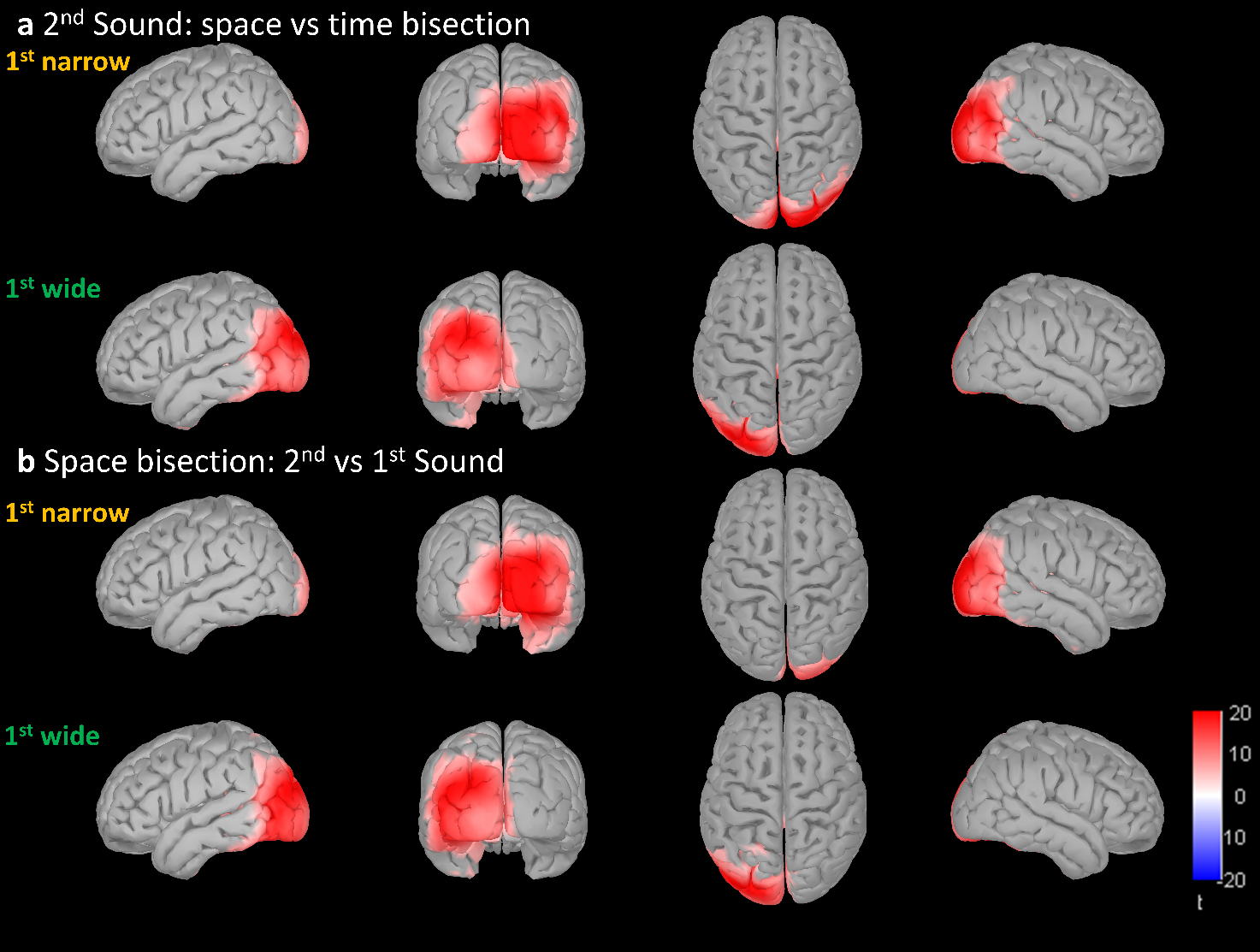


**Supplementary Figure 10**. Second sound during space bisection elicits a specific late activation of contralateral parieto-occipital cortex. Results of pairwise two tailed t-tests performed on average source activity in the (300-500 ms) time window. On each line, different views of the same comparison between cortical activations; from left to right: left, back, dorsal and right. Comparisons were performed (**a**) between space and time bisection tasks after second sound and (**b)** between the second and the first sound within spatial bisection task. Signed values of t are displayed: reddish colors indicate that space bisection (**a)** or second sound (**b**) produced a stronger cortical activation respectively than time bisection or first sound. Only t values corresponding to p<0.0001 after FDR correction are displayed.

# ERP before cleaning procedure


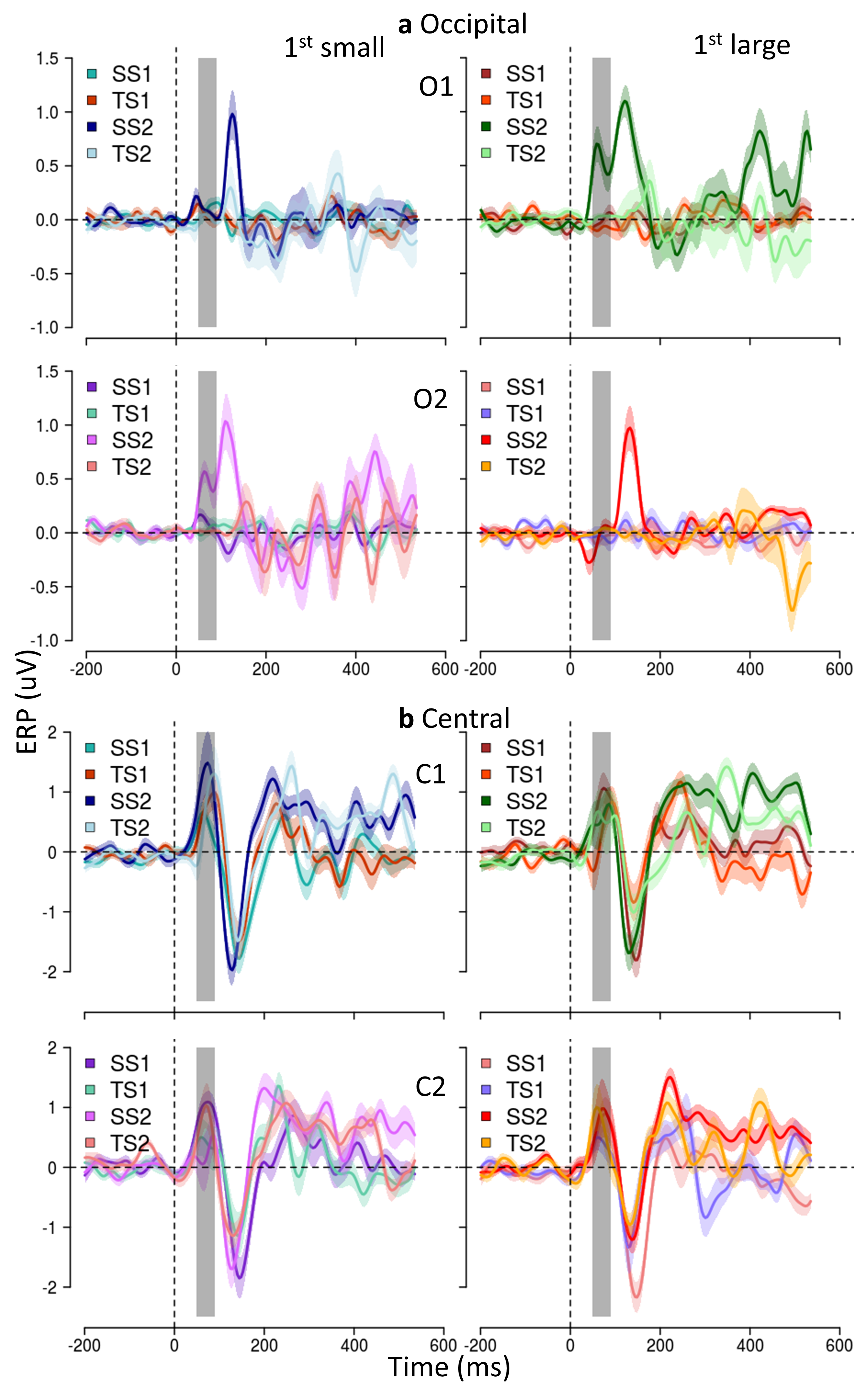


**Supplementary Figure 11.** Physical position of the second sound modulates early response in Occipital but not in Central areas even considering data before cleaning procedure. **a** Occipital areas. ERP (mean ± SEM) in O1, first raw, and in O2, second raw, averaged across subjects. On the left, average of trials in which S2 is presented in the left hemispace (spatial bisection) or with shorter temporal separation from S1 (temporal bisection); on the right, trials in which S2 is presented in the right hemispace or with longer temporal separation from S1. Differently colored curves represent ERP responses to the first and the second sound for temporal (TS1, TS2) and spatial bisection task (SS1, SS2). t = 0 is sound onset. Shaded area delimits early ERP component time window (50-90 ms), commonly corresponding to major visual responses. **b** ERP in C1, first line, and in C2, second line (mean ± SEM). Only in Occipital areas both the early and a later response (starting around 350 ms after the sound) show a contralateral pattern with respect to the second sound and during spatial bisection task. Task related modulations are less evident with respect to cleaned data, due to the worse signal to noise ratio, but still present.


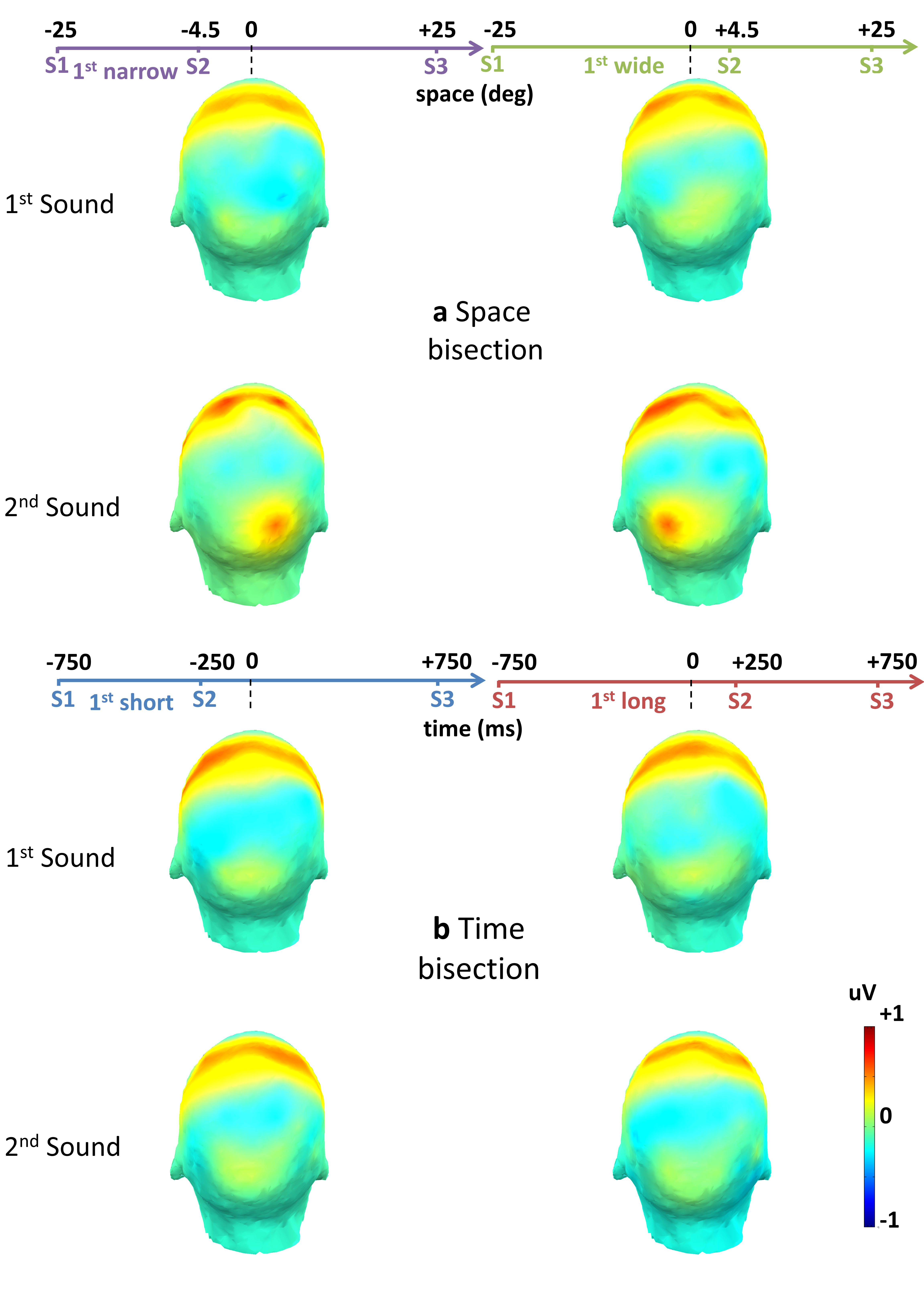


**Supplementary Figure 12.** ERP scalp map in the (50-90 ms) time window averaged across subjects considering data before cleaning procedure. Two strong positivities emerged. One, involving central areas, was not modulated by experimental manipulation. The other, involving parieto-occipital areas, showed a specific contralaterality during space bisection task (**a**) when a narrow (Left) or a wide (Right) first interval respectively corresponded to a second sound in the left or in the right hemifield. During time bisection task (**b**), neither a short nor a long first interval in the time domain could elicit a similar response. The observed contralateral response was specific of the second sound, while absent after the first one. Importantly, no other scalp areas were involved by early positivity. Task related modulations are less evident with respect to cleaned data, due to the worse signal to noise ratio, but still present.


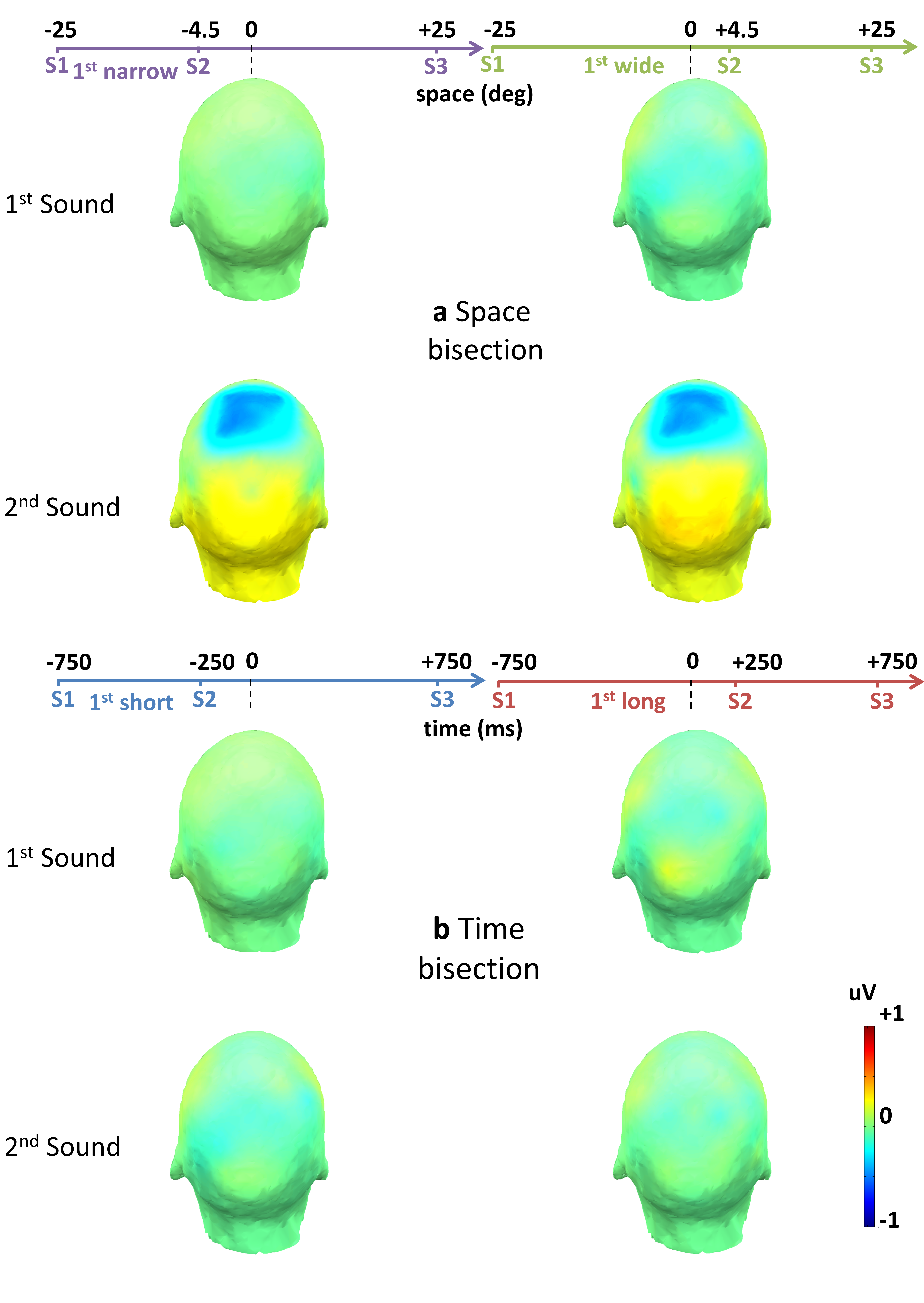


**Supplementary Figure 13.** ERP scalp map in the (110 -160 ms) time window averaged across subjects considering data before cleaning procedure. During space bisection (**a**) the second sound produced a positivity in in parieto-occipital areas, while a negativity in front-central areas. Both positivity and negativity were not modulated by sound position. During time bisection task (**b**), a similar response was missing, as well as after the first sound for both bisection tasks. Task related modulations are less evident with respect to cleaned data, due to the worse signal to noise ratio, but still present.

**
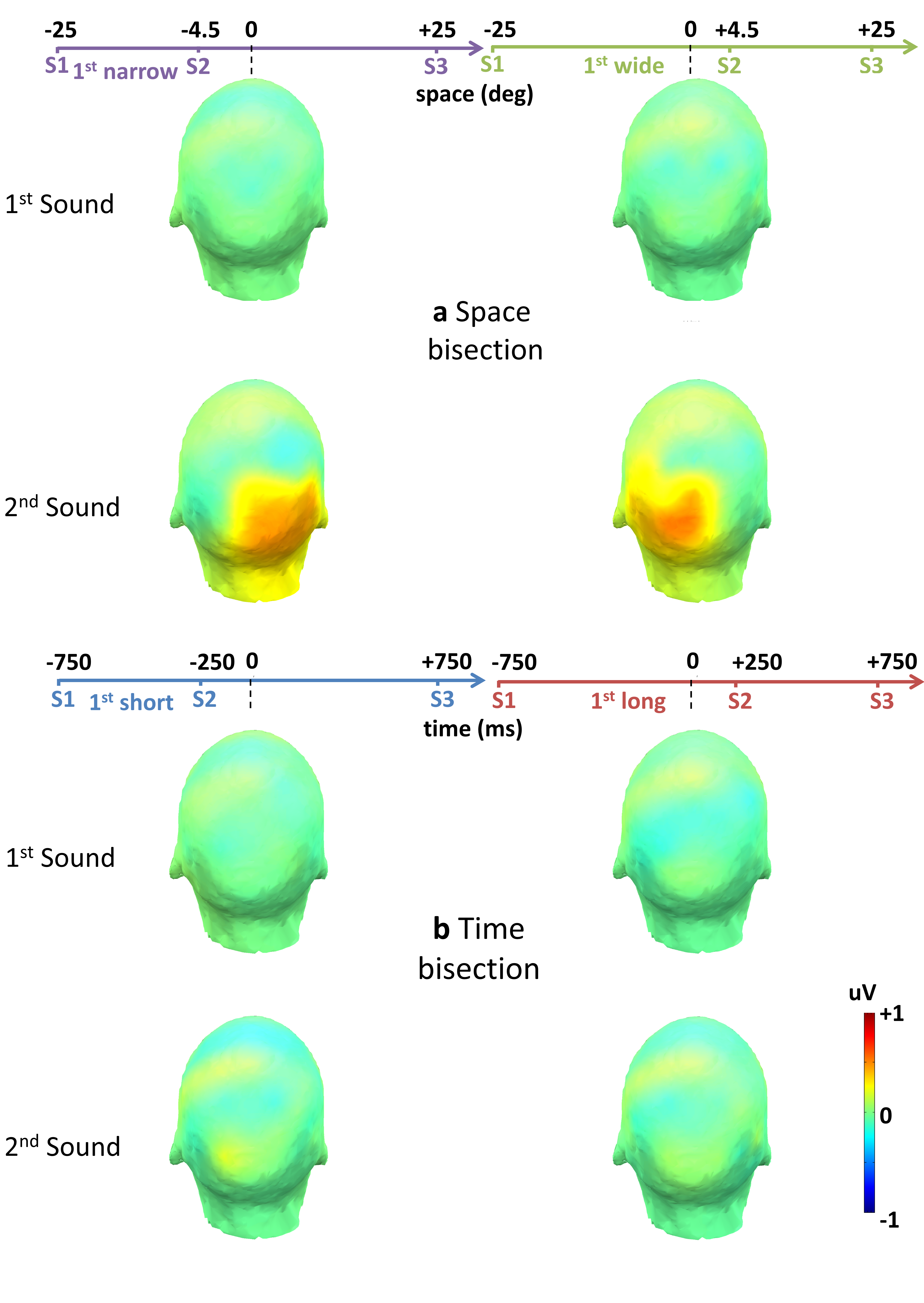
**

**Supplementary Figure 14.** ERP scalp map in the (300-500 ms) time window averaged across subjects considering data before cleaning procedure. During space bisection (**a**) the second sound produced a positivity in in parieto-occipital areas contralateral to sound position, which was absent during temporal bisection (**b**) or, for both bisection tasks, after the first sound. Task related modulations are less evident with respect to cleaned data, due to the worse signal to noise ratio, but still present.
